# Evidence for ancient fractional melting, cryptic refertilization and rapid exhumation of Tethyan mantle (Civrari Ophiolite, NW Italy)

**DOI:** 10.1007/s00410-019-1603-5

**Published:** 2019-08-01

**Authors:** Anders McCarthy, Othmar Müntener

**Affiliations:** 1 Institute of Earth Sciences, University of Lausanne, 1004 Lausanne, Switzerland; 2 Present Address: School of Earth Sciences, University of Bristol, Bristol, BS8 1RJ UK

**Keywords:** Abyssal peridotite, Gabbro, Cryptic refertilization, Western Alps

## Abstract

Western Tethyan peridotites exposed in the European Alps show limited amounts of partial melting and mostly fertile compositions. Here we investigate the Civrari Ophiolite (northwestern Italy), which is composed of depleted spinel-harzburgites and serpentinites associated with MOR-type gabbros and basalts. The ultramafic rocks are unique amongst western Tethyan peridotites, showing homogeneous residual compositions after ~ 15% near-fractional melting, lack of pervasive melt percolation and mineral compositions that indicate high-temperature equilibration ≥ 1200 °C. Clinopyroxene chemistry records some of the lowest abundances of Na_2_O, Ce, and Zr/Hf amongst abyssal peridotites worldwide, suggesting that most abyssal peridotites have been affected by variable degrees of melt retention upon melting or cryptic melt percolation. Locally, cryptic MORB-like melt migration in Civrari peridotites produced orthopyroxene + plagioclase intergrowth around reacted clinopyroxene. These clinopyroxene preserve micron-scale chemical zoning indicating rapid cooling after melt crystallization. ^143^Nd/^144^Nd isotopic data indicate that Civrari mantle rocks, gabbros, and basalts are not in isotopic equilibrium. Civrari spinel-peridotites represent a highly radiogenic endmember amongst Western Tethys depleted spinel-peridotites, which together form a partial melting errochron of 273 Ma ± 24 Ma. Ancient near-fractional melting and cryptic melt–rock reaction cause variations in radiogenic εNd and εHf, leading to isotopic heterogeneity of Western Tethys mantle rocks. Such inherited signatures in mantle rocks are most likely to be preserved along (ultra-)slow-spreading systems and ocean–continent transition zones.

**Electronic supplementary material:**

The online version of this article (10.1007/s00410-019-1603-5) contains supplementary material, which is available to authorized users.

## Introduction

The formation of oceanic lithosphere at mid-ocean ridges was spearheaded by the discovery of mid-ocean ridges (e.g., Heezen et al. 1964) and the characterization of ophiolites as remnant oceanic lithosphere thrust upon continental margins (e.g., Anonymous 1972; Decandia and Elter 1972). Over the last 30 years, the diversity of oceanic spreading systems has been underscored by the discovery of (ultra-)slow spreading systems (e.g., Dick et al. 2003) and magma-poor rifted margins (e.g., Boillot et al. 1980). In these cases, oceanic lithosphere is dominated by exhumed mantle, oceanic core complexes and minor basalts and gabbros, with extension being accommodated by the exhumation of mantle to the ocean floor along detachment faults (e.g., Dick et al. 1981; Tucholke et al. 1998). Field studies have pointed out numerous similarities between modern ultra-slow spreading systems, magma-poor rifted-margins and ophiolites in the Western Tethys (e.g., Lagabrielle and Cannat 1990; Lagabrielle et al. 2015; Manatschal and Müntener 2009).

Constraining the petrological and geochemical characteristics of the convective upper mantle has primarily centered on the study of exhumed mantle domains along rifted margins and (ultra-)slow spreading systems (e.g., Elthon 1992; Dick 1989; Snow et al. 1994; Rampone and Hofmann 2012; Picazo et al. 2016; Warren 2016). Although abyssal peridotites were initially thought to be simple residues of adiabatic decompression fractional melting (Johnson et al. 1990), trace element abundances of clinopyroxene and abundance of plagioclase-bearing peridotites indicate that abyssal peridotites record varying degrees of diffuse melt percolation and melt retention (e.g., Elthon 1992; Dick 1989; Brunelli et al. 2006; Warren et al. 2009). Moreover, isotopic studies of mantle rocks exhumed along rifted margins and (ultra-)slow spreading systems have demonstrated the chemical and isotopic heterogeneity of exhumed mantle rocks at small (< meter) and large scale. These data have been primarily explained as a result of the inefficiency of mantle convection, allowing for ancient (> 1 to 2 Ga) refractory mantle domains to persist in the convective upper mantle (e.g., Tribuzio et al. 2004; Cipriani et al. 2004; Stracke et al. 2011; D’errico et al. 2016). Alternatively, it was proposed that depleted mantle domains along ocean–continent transition zones (OCTs) preserved in Western Tethys ophiolites might reflect “ancient” crust-forming events (Rampone et al. 1998; Müntener et al. 2004; McCarthy and Müntener 2015). However, melt percolation in exhumed mantle domains along OCTs and (ultra-)slow spreading systems (e.g., Müntener et al. 2010; Warren and Shimizu 2010) has the tendency to obscure the original composition of depleted mantle domains, making it difficult to relate specific mantle domains to a particular tectonic setting and magmatic processes.

We present a petrological study of one of the most depleted peridotites in the Western Tethys. The Civrari Ophiolite consists of serpentinites and highly depleted residual high-temperature peridotites. The depleted nature of the Civrari mantle clinopyroxene, affected by near-fractional melting, indicates that residual abyssal peridotites do not reflect simple residues after partial melting. We also show how cryptic MORB-type melt percolation occurs at shallow depth, leading to grain-scale chemical zonation of clinopyroxene and occasional plagioclase and apatite saturation. We discuss the possible causes of highly radiogenic Hf–Nd signatures in mantle rocks and suggest that the lack of isotopic equilibrium between mantle rocks and associated magmatism along (ultra)-slow spreading systems and ocean–continent transition zones might be caused by ancient near-fractional melting events and subsequent accretion to the subcontinental mantle.

## Geological setting

Western Tethys ophiolites found in the European Alps and Apennines represent remnants of an ancient analogue to present day (ultra)-slow spreading environments (Mid-Atlantic Ridge, Gakkel ridge) and OCTs (Iberia-Newfoundland) (e.g., Manatschal and Müntener 2009; Lagabrielle et al. 2015). The majority of exhumed ultramafic rocks consist of heterogeneous subcontinental lithosphere emplaced along ocean–continent transition zones during lithospheric thinning and exhumation in Jurassic times (e.g., Meresse et al. 2012; Rampone and Hofmann 2012; Müntener et al. 2004, Rampone et al. 1998).

The Lanzo massif, located north of Torino, shows a complex history of regional scale refertilization, melt–rock interaction, melt migration in dunite conduits and the formation of extensional shear-zones (Müntener and Piccardo 2003; Piccardo et al. 2007; Kaczmarek and Müntener 2008, 2010; Guarnieri et al. 2012, Sanfilippo et al. 2017), similar to slow-spreading ridges (Hellebrand and Snow 2003) and other Western Tethyan ophiolites (Borghini et al. 2007; Müntener et al. 2010). The presence of an oceanic sedimentary cover (Lagabrielle et al. 1989) coupled with continental rocks (Sesia–Lanzo zone) associated with the Lanzo ultramafic rocks suggests that Lanzo-Sesia might be a high-pressure analogue of an ocean–continent transition zone (Pelletier and Müntener 2006).

**Fig. 1:**
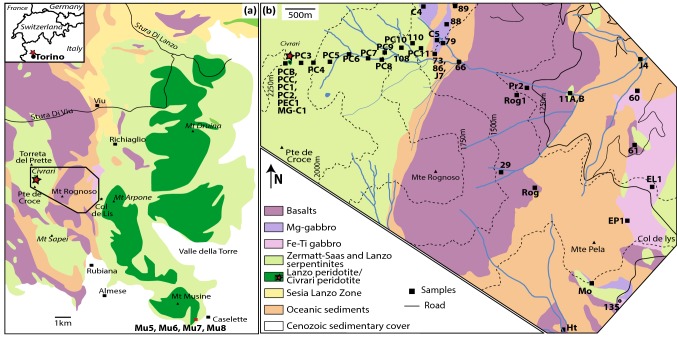
**a** Lanzo-Civrari Ophiolite, Northern Italian Alps modified after Boudier (1978) and Pognante et al. (1986), with location of studied Southern Lanzo gabbros (GPS coordinates: N. 45°6′24, E. 7°28′62) as well as the Monte Civrari peridotites (GPS coordinates: N.45°11′32, E.7°19′53), the latter being represented by a red star. **b** Civrari Ophiolite, with location of samples. Oceanic sediments are Jurassic micaschists, calcschists, quartzites, and ophicarbonates

The Civrari Ophiolite is a sliver of the Piemontese Ophiolites and is located to the West of the Lanzo massif. It consists mainly of serpentinites associated with a thin sedimentary cover of micaschists, calcschists, and quartzites. Field observations show that mafic rocks are composed of variably metamorphic and discontinuous Mg- and Fe–Ti gabbros (Fig. 1). MOR-type metabasalts cover a 2.5 km-long, N–S trending area within the sedimentary cover to the East of the Civrari serpentinites. Most of the Civrari ophiolitic rocks are overprinted by Alpine metamorphism, but locally fresh rocks are preserved.

### Location and sample description

**Fig. 2:**
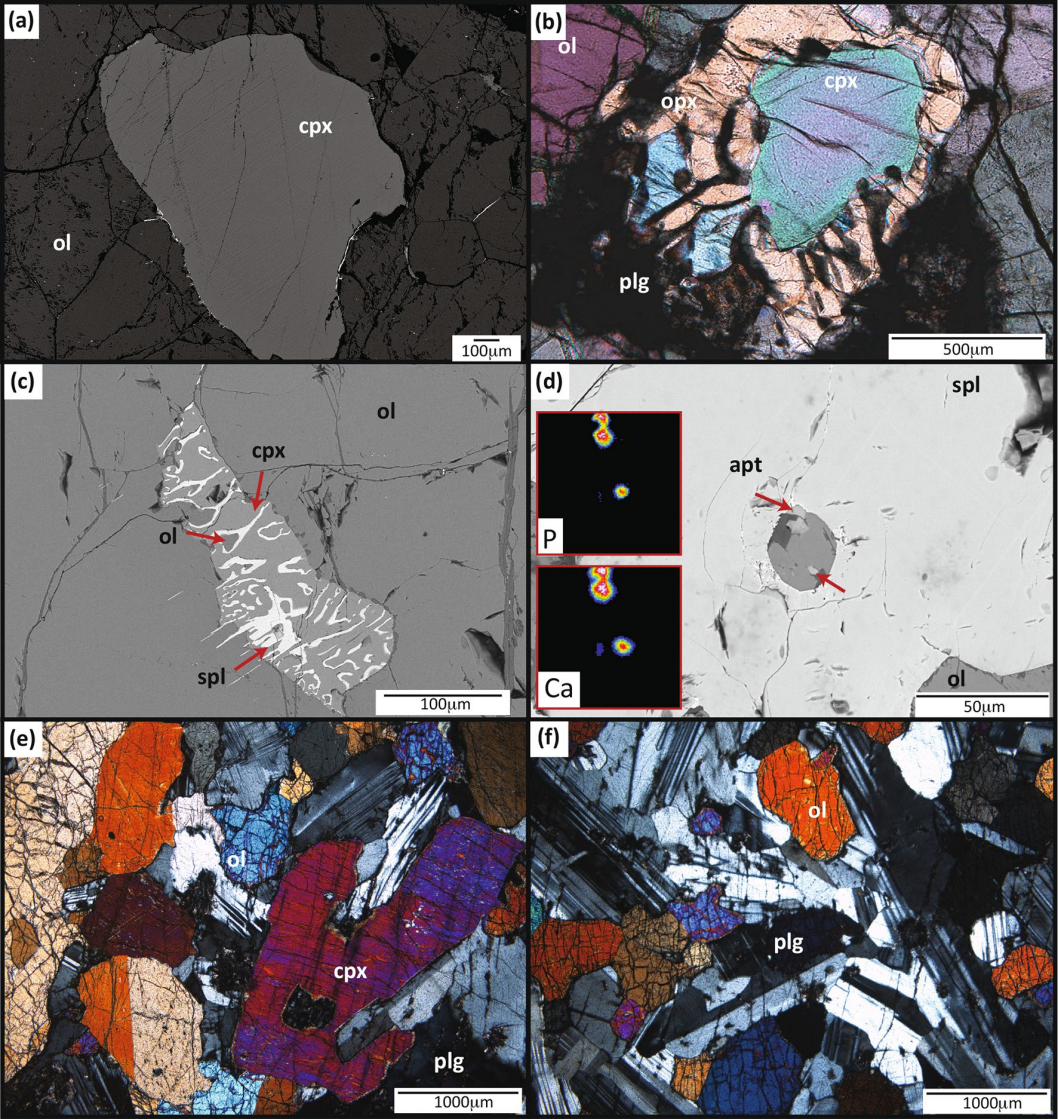
**a** Reactive clinopyroxene (cpx) with rounded, concave rims, showing only minor textural evidence of interaction with percolating MORB-melts. *Ol* olivine; **b** Rounded reactive clinopyroxene with orthopyroxene–plagioclase rims (opx and plg, respectively); **c** interstitial symplectite of Cr-spinel (spl) and clinopyroxene (cpx) between olivine in both reactive and refractory Civrari spinel-peridotites. **d** SEM image of a Cr-spinel with inclusions of 5–10 μm apatite grains (apt). The presence of apatite is highlighted in the red squares, which show P and Ca chemical maps. **e** Gabbroic section of the Civrari dyke, with plagioclase, clinopyroxene and olivine. **f** Troctolite section of the Civrari dyke, with olivine and euhedral plagioclase

The Civrari peridotite consists of a very fresh, 10–15 m-wide lens of a clinopyroxene- and spinel-bearing harzburgite associated with cm–dm-thick gabbro dykes and occasional dm-thick serpentinised pyroxenites. The peridotites can be subdivided into refractory (sample PCB) and reactive harzburgites (sample PC2). The reactive peridotite displays a significant range in clinopyroxene shape and size, from rare 2 mm-wide coarse clinopyroxene (Fig. 2a) to rounded, anhedral and compositionally zoned 200–500 μm clinopyroxene rimmed by spinel or intergrowth of orthopyroxene + plagioclase (Fig. 2b). Clinopyroxene-spinel ± olivine intergrowths are found interstitially in both types of peridotites (Fig. 2c), whereas small (< 10 μm) apatite grains are found exclusively associated with plagioclase around partially dissolved spinel in the reactive peridotite (Fig. 2d). Plagioclase intergrown with orthopyroxene has been fully altered and replaced by a metamorphic assemblage of zeolites, Al-rich chlinochlore, Al–Mg hydroxides, garnet, clinozoisite, diopside and a variety of fine-grained hydrous aluminosilicates.

Basalts and gabbros were analyzed for whole-rock major and trace elements (Fig. 1b). Mg-gabbros are predominantly found at the contact with the Civrari serpentinite and are locally overlain by garnet-bearing quartzites. Isolated bodies of gabbros might be in direct contact with ophicarbonates and serpentinites or Fe–Ti gabbros. Mg-gabbros show locally preserved magmatic textures and preserved magmatic clinopyroxene. Five dm–cm-thick gabbro dykes intruding host peridotites, ranging from primitive olivine gabbros (MG-C1, Mu5, and Mu6) to slightly differentiated gabbros (Mu7, Mu8) were collected (Fig. 1a, b). These gabbros show well-preserved magmatic assemblages of clinopyroxene, plagioclase and olivine. Civrari gabbro MG-C1 is a composite gabbro with a troctolite zone composed of anhedral olivine and euhedral plagioclase grading abruptly into a gabbroic assemblage with olivine, euhedral plagioclase and subhedral to interstitial clinopyroxene (Fig. 2e, f). Lanzo gabbros show mm–cm large euhedral clinopyroxene, anhedral olivine, and interstitial plagioclase.

Fe–Ti gabbros are found as cm–dm thick dykes within deformed serpentinites or as larger, discontinuous bodies (Fig. 1). Two Fe–Ti gabbro dykes (FG-11A and -11B) sampled within the serpentinite show variable mineralogy, with *FG*-*11A* dominated by a rodingite assemblage of small anhedral hydrogrossular, fine-grained epidote, chlorite and rutile (± titanite), and *FG*-*11B* dominated by chlorite, large euhedral magnetite grains and large titanite aggregates.

Mg-gabbros and basalts are predominantly formed of chlorite, tremolite-actinolite, clinozoisite-epidote, plagioclase and titanite coronas around rutile, with local high-pressure relicts preserved (garnet and blue amphibole). Fe–Ti gabbros preserve an eclogite facies assemblage of garnet, omphacite, glaucophane, rutile and quartz, partly retrogressed to greenschist facies.

### Analytical procedures

Whole-rock samples were powdered in an agate mill and dried to ca. 100 °C, with loss on ignition (LOI) determined by heating the sample to 1050 °C for 2 h. Li-tetraborate glasses were made by fusing 1.2 g of dried sample with 6 g of Lithium Tetraborate at 1300° for 3.5 min in Pt-crucibles followed by quenching. Major element compositions were measured on Li-tetraborate glasses and acquired by X-ray fluorescence using a Philips PW 2400 spectrometer at the Institute of Earth Sciences (ISTE), University of Lausanne (UNIL), Switzerland (Table 1). Cr and Zn for ultramafic rocks were measured by X-ray Fluorescence Philips PW 2400 spectrometer on compressed tablets of finely ground rock powder mixed with 20–30% of a cellulose wax. The standards SY-2, NIMN, NIMG, BHVO and UB-N were used as quality control (Govindaraju 1994). Uncertainties in XRF analyses are in the range 0.5 wt% (2*σ*) for major components such as SiO_2_ to 0.01wt% for minor elements. In situ trace element abundances were measured on the flat side of broken off pieces of Li-tetraborate glasses using a quadrupole spectrometer Elan 6100 DRC for mafic rocks and a sector-field Inductively Coupled Plasma Mass Spectrometer ELEMENT2 XR interfaced to a NewWave UP-193 ArF excimer ablation system for ultramafic rocks. Analytical conditions were 10–20 Hz repetition rate and an energy of 160 mJ, which is equivalent to 12 J cm^−2^ with a beam size of 100 microns. The NIST SRM 612 glass standard was used to maximize sensitivity. Three repeat measurements were performed on each glass bead, with average standard deviation and detection limits listed in Table 2. Helium was used as a cell carrier gas. Dwell times for different isotopes range from 10 to 20 ms, employing a peak-hopping mode. Trace element concentrations were determined using CaO previously measured by XRF as an internal standard and NIST SRM 612 as an external standard (Jochum et al. 2011). Data were processed using LAMTRACE software (Jackson 2008).

**Table 1:** Whole-rock major element composition

Sample	Lithology	SiO_2_	TiO_2_	Al_2_O_3_	Fe_2_O_3_	MnO	MgO	CaO	Na_2_O	K_2_O	P_2_O_5_	Cr_2_O_3_	NiO	LOI	Sum	Mg#_Fetot_
PCC	Per.	43.23	0.03	1.66	9.10	0.13	43.30	1.66	0.00	0.00	0.01	0.44	0.29	0.00	99.84	90.41
PCB	Per.	43.61	0.03	1.92	8.63	0.13	41.12	3.38	0.00	0.00	0.01	0.55	0.27	0.32	99.97	90.42
PC2	Per.	41.71	0.02	1.28	9.42	0.13	44.71	1.41	0.00	0.00	0.01	0.37	0.30	− 0.09	99.27	90.39
PC1	Serp.	40.09	0.03	1.58	8.73	0.13	39.15	0.68	0.00	0.00	0.01	0.44	0.26	9.14	100.24	89.88
PC3	Serp.	41.24	0.02	1.77	5.95	0.14	38.60	2.22	0.00	0.00	0.01	0.40	0.25	9.19	99.77	92.78
PC4	Serp.	39.96	0.02	1.54	8.41	0.17	38.25	2.07	0.00	0.00	0.01	0.42	0.25	8.69	99.79	90.01
PC5	Serp.	40.74	0.03	1.49	6.66	0.15	38.62	1.99	0.00	0.00	0.01	0.35	0.22	9.29	99.56	91.99
PC6	Serp.	40.38	0.02	1.85	6.97	0.09	39.22	0.03	0.00	0.00	0.01	0.55	0.27	10.89	100.28	91.77
PC7	Serp.	40.85	0.01	1.79	6.53	0.10	37.94	1.83	0.00	0.01	0.01	0.45	0.28	10.39	100.18	92.01
PC8	Serp.	39.50	0.03	1.48	7.63	0.14	38.79	1.79	0.00	0.00	0.00	0.39	0.27	10.26	100.28	90.97
PC9	Serp.	41.33	0.02	1.73	4.94	0.11	39.78	0.74	0.00	0.00	0.01	0.33	0.27	10.91	100.15	94.10
PC10	Serp.	38.05	0.03	1.66	10.83	0.11	37.52	0.00	0.00	0.00	0.01	0.60	0.27	11.00	100.07	87.28
PC11	Serp.	39.98	0.02	1.61	7.02	0.10	39.00	0.02	0.00	0.00	0.01	0.30	0.27	11.63	99.97	91.67
108	Serp.	40.05	0.02	1.95	6.89	0.11	39.17	0.60	0.00	0.00	0.01	0.46	0.28	10.62	100.17	91.85
110	Serp.	40.76	0.03	1.47	7.29	0.11	38.13	0.07	0.00	0.00	0.01	0.39	0.25	11.45	99.97	91.19
PEC1	Serp.	39.98	0.03	1.88	8.09	0.13	38.89	0.98	0.00	0.00	0.01	0.58	0.26	9.39	100.22	90.49
FG-EP1	Fe–Ti gab	45.60	4.80	9.89	21.97	0.33	5.15	8.48	3.55	0.07	0.25	0.00	0.00	− 0.25	99.9	31.72
FG-EL1	Fe–Ti gab	49.91	2.67	11.71	18.99	0.20	3.54	6.33	4.99	0.04	1.44	0.00	0.00	0.31	100.1	26.96
FG-60	Fe–Ti gab	49.08	3.47	9.55	18.30	0.24	6.13	7.84	3.85	0.14	0.05	0.00	0.00	0.91	99.6	39.90
FG-135	Fe–Ti gab	43.12	9.29	9.71	19.79	0.33	5.17	8.78	3.58	0.03	0.04	0.01	0.01	0.08	100.0	34.08
FG-J4	Fe–Ti gab	44.11	4.42	12.01	21.71	0.38	5.65	8.28	2.82	0.05	0.13	0.02	0.01	0.14	99.7	34.03
FG-11B	Fe–Ti gab	26.31	7.80	10.39	23.43	0.11	19.31	5.57	0.00	0.00	0.02	0.01	0.02	6.97	99.9	62.01
FG-11A	Fe–Ti gab	31.81	6.87	13.11	20.41	0.31	6.39	17.28	0.00	0.01	0.02	0.01	0.01	3.43	99.7	38.28
MG-C4	Mg-gab	47.40	0.34	20.68	3.56	0.07	8.55	15.50	1.47	0.08	0.03	0.14	0.02	2.49	100.3	82.64
MG-J4	Mg-gab	50.83	0.61	3.93	14.18	0.33	16.56	10.08	0.58	0.03	0.19	0.22	0.11	2.46	100.1	69.82
MG-C1	Mg-gab	47.93	0.13	17.84	4.27	0.08	13.79	13.65	1.28	0.04	0.01	0.27	0.06	0.41	99.8	86.48
MG-66	Mg-gab	48.14	0.04	3.62	9.04	0.15	17.67	14.25	0.39	0.02	0.01	0.17	0.19	6.18	99.9	79.47
MG-86	Mg-gab	47.85	0.33	16.33	7.73	0.21	15.36	4.15	1.71	0.00	0.06	0.04	0.04	5.86	99.7	79.75
MG-89p	Mg-gab	47.14	0.34	17.87	7.51	0.13	11.25	8.19	2.83	0.09	0.05	0.07	0.06	3.93	99.5	74.79
MG-89g	Mg-gab	45.62	0.41	18.51	6.78	0.10	12.12	8.99	0.71	1.19	0.12	0.31	0.04	4.66	99.6	77.99
MG-88	Mg-gab	42.98	0.09	22.51	3.43	0.07	9.94	11.57	1.80	1.08	0.01	0.03	0.06	6.20	99.8	85.16
MG-J7	Mg-gab	45.67	0.38	11.66	6.15	0.16	13.53	10.64	0.00	0.78	0.02	0.30	0.07	10.55	99.9	81.33
MG-79	Mg-gab	45.51	0.07	22.43	3.64	0.07	10.61	8.79	2.71	0.54	0.01	0.04	0.08	4.60	99.1	85.23
MG-73	Mg-gab	47.60	0.09	21.49	4.12	0.06	12.46	3.78	3.89	0.78	0.01	0.03	0.06	5.34	99.7	85.71
MG-Mo	Mg-gab	47.92	0.52	16.09	5.99	0.09	6.90	17.89	2.37	0.05	0.01	0.02	0.02	2.28	100.2	69.53
MG-C5	Mg-gab	43.20	0.08	21.92	4.39	0.07	12.37	10.99	0.99	0.26	0.01	0.02	0.08	5.06	99.4	84.79
Rog1	MORB	43.58	1.14	15.16	7.33	0.15	6.09	15.44	3.12	0.07	0.16	0.05	0.02	7.17	99.5	62.19
Pr2	MORB	46.69	1.15	15.65	8.27	0.12	6.60	14.48	2.32	0.40	0.19	0.05	0.01	4.03	100.0	61.26
29	MORB	48.35	1.26	16.96	9.26	0.16	9.18	8.49	2.78	0.05	0.12	0.06	0.02	3.42	100.1	66.27
Rog	MORB	49.93	1.40	15.55	9.72	0.17	8.56	7.76	3.61	0.05	0.16	0.03	0.01	2.88	99.8	63.55
Ht	MORB	48.86	1.22	16.38	8.94	0.15	9.32	8.11	3.20	0.40	0.08	0.06	0.02	3.07	99.8	67.39
61	MORB	48.77	1.59	15.81	10.03	0.16	6.38	12.78	1.73	0.06	0.18	0.05	0.02	2.17	99.7	55.75

Note that the slightly higher CaOwt% abundance in sample PCB is related to diffuse fine-grained calcite and serpentinite

*Per.* peridotite, *serp* serpentinite, *gab* gabbro, *MORB* Mid-Ocean Ridge basalt

**Table 2:** Trace element composition of whole rocks

ppm	PCC	PCB	PC2	PC1	PC3	PC4	PC5	PC6	PC7	PC9	PC10	PC11	108	110	PEC1	FG-EP1	FG-EL1	FG-60	FG-135	FG-J4	FG-11B	FG-11A	MG-C4
	Per.	Per.	Per.	Serp.	Serp.	Serp.	Serp.	Serp.	Serp.	Serp.	Serp.	Serp.	Serp.	Serp.	Serp.	Fe–Ti gab	Fe–Ti gab	Fe–Ti gab	Fe–Ti gab	Fe–Ti gab	Fe–Ti gab	Fe–Ti gab	Mg-gab
Sc	13.5	17.2	7.09	11.49	11.87	11.87	11.76	13.05	9.20	13.75	12.43	12.80	12.61	12.97	12.83	50.2	34.1	44.8	70.5	55.8	45.7	56.2	22.5
V	69.2	90.3	35.86	64.83	50.97	58.33	43.72	64.89	53.79	62.30	88.85	72.57	65.78	61.68	66.66	412.7	90.3	575.5	1339.6	1527.8	651.7	816.1	114.0
Cr	2371	2956.1	1860.3	2659	2482	2725	2234	3404	2634	1975	3807	1822	2878	2554.9	2949.4	50.3	71.2	51.1	53.1	86.4	84.4	48.3	984.9
Zn	45.7	43.3	46.1	42.4	37.5	44.8	40.2	40.3	38.1	40.7	43.7	38	42	49.3	41.6	146.8	139.2	125.5	49.8	99.4	62.2	88.0	18.7
Co	111	106	44	117	100	106	105	105	113	111	115	151	148	110	114	38.0	17.0	45.3	63.7	47.8	63.5	54.8	23.1
Ni	2265	2032	–	2311	1927	1952	1720	2296	2223	2302	2327	2631	2265	2160	2291	9.7	6.7	26.9	47.3	74.3	177.8	98.7	170.2
Cu	24.0	17.8	7.42	11.82	14.22	17.72	11.62	21.50	14.50	13.91	8.55	25.45	6.47	17.12	18.58	20.3	11.8	21.9	50.4	174.4	8.2	36.8	15.7
Mo	37.1	36.3	1.26	0.12	0.15	0.49	0.82	0.25	0.16	1.20	0.15	0.74	0.79	0.67	0.26	–	–	–	–	–	–	–	–
Rb	0.1	0.1	0.04	0.48	0.40	0.16	0.21	0.36	0.45	0.33	0.47	0.10	0.09	0.17	0.25	1.66	1.09	7.04	1.15	1.15	0.13	1.07	1.06
Sr	0.229	0.144	0.222	0.607	0.773	0.621	0.569	1.681	0.677	0.400	0.322	0.718	0.287	0.889	1.860	47.56	134.10	31.64	26.23	21.46	20.83	612.63	315.38
Y	0.600	1.192	0.505	5.112	0.798	0.859	0.714	0.612	0.475	0.593	0.827	0.442	0.287	0.384	0.573	41.16	103.14	35.67	29.78	26.84	74.24	34.99	7.55
Zr	0.219	0.254	0.162	17.295	1.330	0.295	0.478	0.269	0.323	0.676	0.431	0.708	0.743	0.516	0.575	76.19	95.66	94.65	110.33	56.39	135.48	90.46	16.81
Nb	b.d.	b.d.	0.009	0.034	0.018	0.016	0.028	0.033	0.026	0.098	0.033	0.021	0.008	0.141	0.031	3.42	4.33	2.09	2.49	0.95	5.60	3.12	0.27
Cs	0.01	b.d.	b.d.	b.d.	b.d.	b.d.	b.d.	0.02	0.02	b.d.	0.02	b.d.	b.d.	b.d.	0.02	0.09	0.06	0.26	0.07	0.03	0.02	0.20	0.20
Ba	2.00	2.14	0.13	4.33	2.50	2.46	2.34	2.34	2.14	2.61	2.55	3.12	1.87	2.08	2.53	5.07	16.19	25.33	8.44	3.15	0.86	13.45	6.65
La	0.01	b.d.	b.d.	0.02	0.01	0.01	0.01	0.03	0.01	0.02	0.01	0.04	0.01	0.03	0.01	2.12	12.72	1.06	0.74	0.75	7.57	1.96	0.73
Ce	0.02	0.03	0.02	0.05	0.03	0.03	0.03	0.47	0.55	0.04	0.37	0.09	0.03	0.10	0.04	8.61	45.71	6.01	2.52	2.45	25.98	7.00	2.05
Pr	b.d.	b.d.	b.d.	0.01	b.d.	0.00	b.d.	0.01	0.01	0.01	b.d.	0.01	0.01	0.01	0.01	1.78	8.38	1.26	0.42	0.57	4.05	1.38	0.44
Nd	b.d.	b.d.	0.01	0.05	0.06	0.03	0.04	0.05	0.01	0.07	b.d.	0.06	0.02	0.06	0.02	11.21	48.33	7.75	2.88	3.76	21.84	8.09	2.21
Sm	b.d.	b.d.	0.02	0.04	0.03	0.02	0.04	0.02	0.00	b.d.	b.d.	0.02	b.d.	0.03	0.01	4.77	15.13	3.10	1.17	2.16	7.60	3.34	0.83
Eu	b.d.	0.02	0.01	b.d.	b.d.	b.d.	b.d.	0.01	0.01	b.d.	b.d.	0.01	b.d.	0.01	0.01	1.97	4.60	1.40	0.67	1.01	4.90	1.93	0.46
Gd	b.d.	0.11	0.04	0.07	0.05	0.04	b.d.	0.04	0.04	b.d.	b.d.	0.04	0.03	0.04	0.04	6.64	19.42	4.92	3.30	3.90	10.42	5.23	1.11
Tb	0.01	0.02	0.01	0.01	0.01	0.01	0.01	0.01	0.01	0.01	0.01	0.01	0.00	0.01	0.01	1.08	3.03	0.91	0.69	0.70	1.81	0.92	0.21
Dy	0.08	0.16	0.09	0.11	0.12	0.12	0.09	0.10	0.05	0.07	0.10	0.10	0.03	0.07	0.08	7.71	19.65	6.52	5.40	4.90	12.43	6.47	1.52
Ho	0.02	0.04	0.02	0.03	0.03	0.03	0.03	0.02	0.02	0.02	0.03	0.02	0.01	0.02	0.02	1.58	3.89	1.38	1.13	1.03	2.66	1.33	0.29
Er	0.08	0.14	0.07	0.12	0.11	0.12	0.11	0.08	0.06	0.07	0.09	0.07	0.03	0.05	0.09	4.38	10.15	4.00	3.36	2.88	8.10	3.80	0.79
Tm	0.02	0.03	0.01	0.02	0.02	0.02	0.02	0.02	0.01	0.02	0.02	0.02	0.01	0.01	0.02	0.63	1.35	0.61	0.50	0.41	1.23	0.54	0.14
Yb	0.12	0.20	0.08	0.16	0.19	0.15	0.20	0.10	0.10	0.11	0.11	0.11	0.10	0.07	0.14	4.21	8.46	4.21	3.43	2.67	8.41	3.86	0.82
Lu	0.02	0.03	0.02	0.03	0.03	0.02	0.03	0.02	0.02	0.02	0.02	0.02	0.02	0.02	0.02	0.61	1.22	0.64	0.53	0.38	1.23	0.56	0.12
Hf	b.d.	b.d.	0.01	0.04	b.d.	0.01	0.02	b.d.	0.02	0.02	0.01	0.02	0.03	0.01	0.01	2.09	2.46	2.55	2.80	1.58	3.63	2.53	0.51
Ta	0.00	b.d.	0.00	0.02	0.01	0.01	0.02	0.01	0.02	0.10	0.01	b.d.	0.01	0.01	0.02	0.26	0.33	0.17	0.20	0.06	0.36	0.25	0.02
Pb	0.38	0.91	0.07	0.49	0.43	0.17	0.22	0.86	1.17	0.29	0.52	0.25	0.16	0.24	0.11	0.84	0.61	1.01	0.63	0.51	0.36	2.82	0.61
Th	b.d.	b.d.	0.00	0.01	0.01	0.01	0.01	0.01	0.01	0.01	0.01	0.03	0.01	0.05	0.01	0.04	0.12	0.04	0.07	0.03	0.13	0.05	0.03
U	b.d.	0.01	0.00	0.01	0.01	0.01	0.01	0.01	0.01	0.02	0.01	0.04	0.00	0.02	0.01	0.04	0.12	0.03	0.16	0.03	0.08	0.03	0.08
W	0.06	b.d.	0.07	0.22	0.17	0.19	0.36	0.13	0.16	0.35	0.15	0.32	0.04	0.12	0.19	–	–	–	–	–	–	–	–

ppm	MG-J4	MG-C1	MG-66	MG-86	MG-89g	MG-88	MG-J7	MG-79	MG-73	MG-Mo	MG-C5	Rog1	Pr2	29	Rog	Ht	61
	Mg-gab	Mg-gab	Mg-gab	Mg-gab	Mg-gab	Mg-gab	Mg-gab	Mg-gab	Mg-gab	Mg-gab	Mg-gab	MORB	MORB	MORB	MORB	MORB	MORB
Sc	21.2	25.9	8.1	10.8	34.3	5.3	19.1	3.6	4.5	2.3	4.7	33.2	33.0	36.5	36.8	35.5	36.7
V	218.0	96.3	43.3	96.7	119.3	31.1	92.0	29.8	26.4	0.6	35.2	222.3	231.1	223.0	273.0	256.5	285.3
Cr	1725.0	2014.8	1311.8	355.9	2274.5	275.3	2303.1	357.3	266.3	207.6	150.4	387.9	372.6	475.3	264.4	451.2	382.3
Zn	195.6	16.6	23.2	52.9	25.1	19.2	54.2	23.8	21.8	31.5	23.3	54.2	44.7	54.0	151.3	70.7	57.7
Co	74.5	41.4	92.2	42.6	38.1	33.2	40.7	41.5	36.2	31.9	40.5	35.9	33.0	40.5	28.0	39.2	35.8
Ni	905.3	462.0	1542.8	305.9	288.8	445.7	587.7	601.5	482.9	181.9	588.4	119.5	109.4	139.3	84.2	139.1	120.0
Cu	19.5	77.0	210.5	37.2	10.2	24.1	95.8	17.5	7.4	36.6	32.8	51.7	52.7	71.9	28.5	49.3	56.9
Mo	–	–	–	–	–	–	–	–	–	–	–	–	–	–	–	–	–
Rb	0.63	0.99	0.19	0.38	15.27	8.32	25.89	6.81	9.92	0.94	3.22	1.72	10.72	0.98	0.90	3.22	1.70
Sr	13.83	100.70	59.63	125.19	155.38	263.67	124.17	233.32	75.55	335.76	135.97	159.99	170.77	119.42	130.71	147.66	123.45
Y	29.16	3.58	1.52	12.44	21.85	3.12	8.20	1.68	1.72	18.08	6.31	25.15	22.86	25.01	29.64	25.14	35.22
Zr	74.59	2.47	1.68	20.93	94.92	3.54	21.08	4.75	4.96	18.18	18.73	71.12	81.53	89.70	90.69	70.26	110.83
Nb	0.61	0.03	0.05	0.47	0.77	0.08	1.02	0.07	0.07	0.29	0.29	1.11	1.41	1.82	1.60	1.50	1.71
Cs	0.05	0.07	0.02	0.04	1.57	0.93	1.20	0.73	0.64	0.05	1.61	0.05	0.19	0.03	0.04	0.27	0.09
Ba	3.80	0.53	0.36	2.94	24.50	33.35	30.59	19.22	28.23	8.01	15.68	6.76	9.75	5.81	11.89	17.32	4.14
La	1.89	0.11	0.05	1.71	2.27	0.47	0.87	0.40	0.39	1.21	1.00	2.31	2.58	3.54	3.27	2.49	3.56
Ce	6.72	0.40	0.16	5.34	7.25	1.34	2.57	0.77	1.04	2.84	2.34	7.93	11.46	12.01	10.38	8.02	11.99
Pr	1.24	0.07	0.03	0.85	1.25	0.20	0.42	0.12	0.15	0.63	0.42	1.35	1.45	1.78	1.79	1.42	2.03
Nd	7.34	0.61	0.16	4.75	6.95	1.12	2.25	0.63	0.73	3.60	2.00	7.88	8.07	9.30	9.51	7.93	11.52
Sm	2.88	0.29	0.06	1.69	2.32	0.29	0.71	0.23	0.20	1.43	0.62	2.84	2.73	3.01	3.27	2.60	3.81
Eu	1.21	0.22	0.05	0.81	0.86	0.28	0.38	0.17	0.26	0.81	0.52	0.99	0.98	1.15	1.23	1.09	1.38
Gd	4.34	0.50	0.10	2.05	3.18	0.34	1.03	0.23	0.21	2.67	0.81	3.50	3.24	3.77	4.40	3.94	5.18
Tb	0.71	0.09	0.02	0.36	0.57	0.07	0.19	0.04	0.05	0.41	0.15	0.59	0.58	0.63	0.74	0.63	0.86
Dy	5.10	0.72	0.22	2.22	3.93	0.47	1.40	0.26	0.30	3.13	0.93	4.35	3.97	4.41	5.29	4.48	6.29
Ho	1.07	0.14	0.05	0.47	0.84	0.10	0.29	0.06	0.06	0.67	0.22	0.93	0.81	0.93	1.14	0.96	1.34
Er	3.00	0.39	0.17	1.29	2.44	0.33	0.90	0.18	0.20	1.87	0.54	2.69	2.32	2.65	3.18	2.81	3.84
Tm	0.42	0.05	0.03	0.18	0.35	0.04	0.14	0.03	0.02	0.26	0.12	0.38	0.37	0.38	0.46	0.40	0.57
Yb	3.04	0.38	0.23	1.13	2.41	0.37	1.02	0.17	0.17	1.64	0.68	2.78	2.22	2.52	3.13	2.73	3.63
Lu	0.48	0.06	0.03	0.17	0.34	0.04	0.17	0.02	0.03	0.23	0.11	0.40	0.35	0.38	0.46	0.40	0.57
Hf	1.69	0.12	0.04	0.61	2.47	0.08	0.62	0.12	0.18	0.55	0.53	1.84	1.85	2.08	2.31	1.96	2.84
Ta	0.05	0.01	0.01	0.03	0.05	0.01	0.05	0.01	0.01	0.02	0.07	0.11	0.13	0.12	0.11	0.10	0.12
Pb	0.78	0.21	0.57	1.21	0.92	0.41	1.80	0.51	0.31	0.96	0.42	0.36	0.57	0.61	1.00	0.78	0.81
Th	0.06	0.01	0.01	0.07	0.06	0.01	0.17	0.02	0.01	0.03	0.08	0.08	0.10	0.13	0.12	0.09	0.11
U	0.02	0.01	0.05	0.23	0.05	0.03	0.06	0.02	0.05	0.11	0.08	0.46	0.11	0.11	0.07	0.10	0.10
W	–	–	–	–	–	–	–	–	–	–	–	–	–	–	–	–	–

ppm	Det. limit ppm	Det. limit ppm	Average 2σ (%)	Average 2σ (%)	SRM612	SRM612
	Ultramafic rocks	Mafic rocks	Mafic rocks	Ultramafic rocks	Average (*n* = 32)	2*σ*
Sc	0.155	0.111	3.37	7.4	41.22	0.57
V	0.063	0.063	2.80	3.8	39.39	0.64
Cr	1	2.550	7.15	–	40.05	6.81
Zn	1	0.327	7.45	–	38.08	2.83
Co	0.056	0.019	3.81	3.2	35.07	1.30
Ni	1.544	3.059	11.07	3.2	38.53	6.88
Cu	0.172	0.210	15.29	9.8	36.87	1.28
Mo	0.059	–	–	27.6	38.46	0.62
Rb	0.027	0.012	8.63	41.2	31.76	0.44
Sr	0.019	0.005	2.57	23.5	76.47	0.93
Y	0.011	0.006	3.70	6.7	38.41	0.55
Zr	0.132	0.008	4.05	27.8	36.14	0.81
Nb	0.011	0.006	15.89	41.2	38.22	0.77
Cs	0.012	0.005	33.70	71.2	41.82	0.54
Ba	0.049	0.028	10.27	13.3	37.90	0.55
La	0.004	0.004	9.06	56.2	35.92	0.71
Ce	0.006	0.004	7.22	29.5	38.51	0.59
Pr	0.005	0.004	13.11	83.2	37.32	0.49
Nd	0.029	0.027	13.94	23.8	35.39	0.78
Sm	0.025	0.026	16.50	56.3	36.87	0.50
Eu	0.008	0.005	13.92	79.8	34.59	0.51
Gd	0.030	0.024	16.41	50.8	37.11	0.70
Tb	0.003	0.003	15.77	60.4	36.07	0.49
Dy	0.014	0.017	13.83	36.7	36.12	0.68
Ho	0.003	0.003	13.44	18.7	38.03	0.76
Er	0.014	0.013	9.50	17.8	37.59	0.59
Tm	0.004	0.004	16.49	47.2	37.71	0.74
Yb	0.011	0.023	13.10	28.4	40.12	0.78
Lu	0.004	0.005	16.60	32.2	37.87	0.63
Hf	0.015	0.010	15.87	25.3	34.92	0.61
Ta	0.007	0.006	24.67	42.7	39.94	0.72
Pb	0.020	0.010	11.31	14.6	39.12	1.24
Th	0.003	0.003	27.68	54.1	37.39	0.83
U	0.003	0.003	32.76	29.1	37.31	0.63
W	0.026	–	–	38.2	39.72	0.88

Representative mineral compositions were measured using a JEOL 8200 Superprobe (ISTE, UNIL) at 15nA, 15 kV with a spot size of 3 μm. The standards used were natural and synthetic silicates or oxides. Na and K were measured in first positions due to possible diffusion. Counting time was 16 s and 8 s on the peak and backgrounds, respectively, for Na and K, and 30 s and 15 s for other elements. Measurements were corrected with the phi-rho-Z matrix correction method (Armstrong 1995). In situ trace element abundances were analyzed on a sector-field Inductively Coupled Plasma Mass Spectrometer ELEMENT2 XR interfaced to a NewWave UP-193 ArF excimer ablation system using a 100–75 μm spot size, 15–20 Hz repetition rate, equivalent to a 6 J/cm^2^ on-sample energy density. Sensitivity was maximized using the NIST SRM 612 glass standard (La^139+^= > 2.5 × 10^6^ c.p.s., Th^232+^= > 2.5 × 10^6^ c.p.s.). The NIST SRM612 glass standard was used during analysis and SiO_2_ or CaO, measured previously on the microprobe, were used as internal standards. Data were processed using LAMTRACE (Jackson 2008).

Sr, Nd, and Pb isotope ratios were measured at the University of Geneva following the protocol and analytical conditions outlined in Chiaradia et al. (2011). Mineral separates were handpicked using a grain fraction between 250 and 100 μm. Mineral separates from samples Mu5, 6, and 7 were leached to remove any possible alteration following the method of Rampone et al. (1998). 100–200 mg of whole-rock powders and mineral separates were leached to remove any secondary minerals with 3 M HCl prior to being rinsed with deionised water and dissolved in Teflon vials containing 4 ml of concentrated HF and 1 ml HNO_3_ 15 M at 140 °C on a hot plate for 7 days. Samples were then dried down and brought back up in 3 ml HNO_3_ 15 M prior to being dried down again. Sr, Nd, and Pb separation was carried out using cascade columns with Sr-spec, TRU-spec and Ln-spec resins following a modified method after Pin et al. (1994). Pb was further purified with a AG-MP1-M anion exchange resin in hydrobromic medium. Sr, Nd, and Pb isotope ratios were measured on a Thermo TRITON mass spectrometer on Faraday cups in static mode at the University of Geneva. Pb was loaded on Re filaments using the silica gel technique and all samples (and standards) were measured at a pyrometer-controlled temperature of 1450 °C. Pb isotope ratios were corrected for instrumental fractionation by a factor of 0.10% per amu based on more than 100 measurements of the SRM981 standard and using the standard values of Todt et al. (1996). External reproducibility of the standard ratios is 0.08% for ^206^Pb/^204^Pb, 0.12% for ^207^Pb/^204^Pb and 0.16% for ^208^Pb/^204^Pb. Sr was loaded on single Re filaments with a Ta oxide solution and measured at a pyrometer-controlled temperature of 1480 °C in static mode using the virtual amplifier design to cancel out biases in gain calibration among amplifiers. ^87^Sr/^86^Sr values were internally corrected for fractionation using a ^88^Sr/^86^Sr value of 8.375209. Raw values were further corrected for external fractionation by a value of + 0.03‰, determined by repeated measurements of the SRM987 standard (^87^Sr/^86^Sr = 0.710250). External reproducibility of the ^87^Sr/^86^Sr ratio for the SRM987 standard is 7 ppm. Nd was loaded on double Re filaments with 1 M HNO_3_ and measured in static mode with the virtual amplifier design. ^143^Nd/^144^Nd values were internally corrected for fractionation using a ^146^Nd/^144^Nd value of 0.7219 and the ^144^Sm interference on ^144^Nd was monitored on the mass ^147^Sm and corrected using a ^144^Sm/^147^Sm value of 0.206700. Raw values were further corrected for external fractionation by a value of + 0.03‰, determined by repeated measurements of the JNdi-1 standard (^143^Nd/^144^Nd = 0.512115: Tanaka et al. 2000). External reproducibility of the JNdi-1 standard is < 5 ppm.

## Results

### Bulk rock major elements

**Fig. 3:**
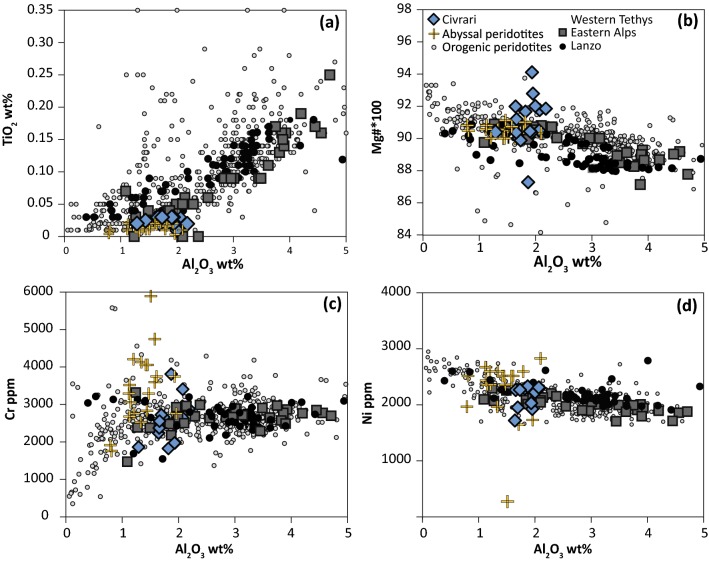
Whole-rock composition of Civrari ultramafic rocks. Data for comparison: Eastern Alps compositions include Malenco, Lower and Upper Platta compositions from Müntener et al. (2010); Lanzo peridotites are from Bodinier (1988), Piccardo et al. (2007) and Kaczmarek and Müntener (2010). Abyssal peridotites from Dick (1989), Brandon et al. (2000) and Stephens (1997); Orogenic peridotite compilation is from Huang et al. (2013)

Peridotites and serpentinites are refractory, as shown by both homogenous and very low TiO_2_ (< 0.05 wt%) and Al_2_O_3_ (< 2.5 wt%) (Fig. 3a). Mg# range from 0.87 to 0.94 and Cr concentrations from 1500–4000 ppm (Fig. 3b). Fe contents of non-serpentinised peridotites are consistent with other ultramafic suites (Fe_2_O_3_ = 8.63–9.42 wt%), with the narrow compositional field of Civrari ultramafic rocks mirroring the compositional field of abyssal peridotites (Fig. 3).

**Fig. 4:**
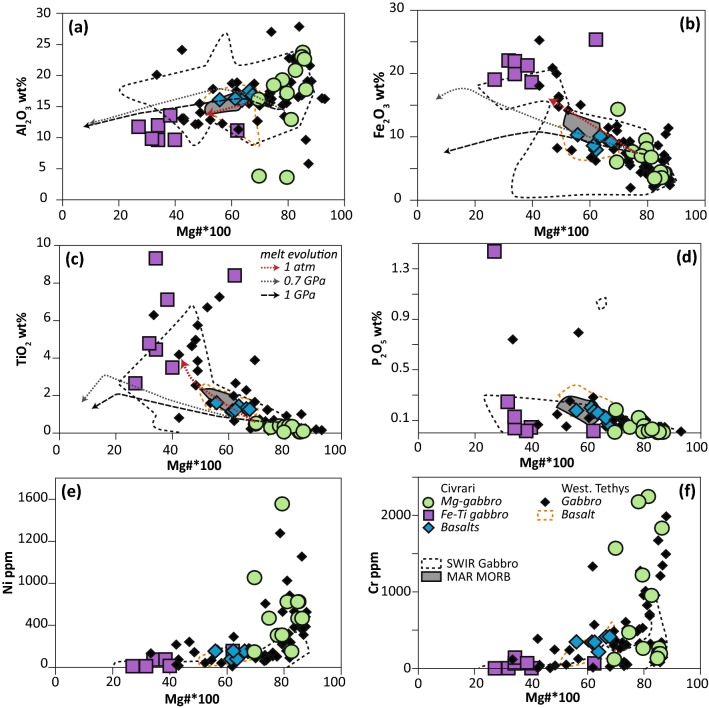
Whole-rock composition of Civrari mafic lithologies. Data for comparison: Western Tethys gabbros and MORBs from Lanzo (Kaczmarek et al. 2008), External Ligurides (Montanini et al. 2008), Platta (Desmurs et al. 2002) and Montgenèvre Ophiolite (Chalot-Prat, 2005). Southwest Indian Ridge (SWIR) gabbros from ODP Leg 176 (Niu et al. 2002) and Mid-Atlantic Ridge (MAR) MORB from Bryan et al. (1981). 1 Gpa and 0.7 Gpa crystal fractionation trends for major elements of dry tholeiitic basalts from Villiger et al. (2004, 2007), and 1 atm trend follows 1 atm experimental data from the literature and compiled in Villiger et al. (2007)

Fe–Ti gabbros are heterogeneous (Fig. 4), with Mg# varying between 27 and 40, with one sample reaching an Mg# of 62. FeO_total_ and TiO_2_ range between 16.7–22.7 wt% and 2.7–9.3 wt%, respectively. Mg-gabbros are heterogeneous, with Mg# ranging from 69.5 to 86. Al_2_O_3_ varies mostly within 16.4–24 wt%, with two samples at 4 wt%. CaO ranges from 4 to 18.3 wt%.

Metabasalts are more homogeneous than gabbros, with Mg# ranging between 56 and 67, similar to MORB (Gale et al. 2013), whilst Al_2_O_3_ varies between 16 and 17.5 wt%. These basalts show slightly higher abundances of TiO_2_ (1.2–1.6 wt%) and P_2_O_5_ (reaching 0.2 wt%) than Mg-gabbros (Fig. 4).

### Bulk rock trace elements

**Fig. 5:**
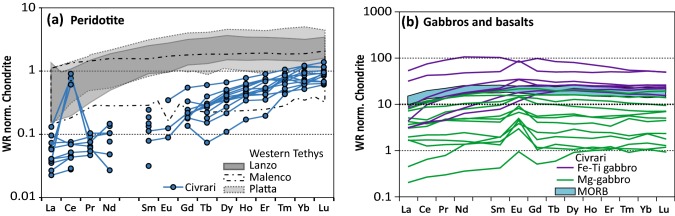
Whole-rock REE normalized to chondrite of **a** peridotites and serpentinites; **b** gabbros and basalts. Data for comparison: Lanzo data from Kaczmarek and Müntener (2010), Malenco and Platta data from Müntener et al. (2010). Chondrite and Primitive Mantle from McDonough and Sun (1995)

REE patterns of ultramafic rocks (Fig. 5) display a strong fractionation in Light-REE (La_N_/Yb_N_ = 0.017–0.23) and Middle-REE (Sm_N_/Yb_N_ = 0.07–0.22) (*N* = normalized to Chondrite-C1, after McDonough and Sun 1995). All samples show fractionated REE patterns (La_N_/Sm_N_ between 0.2 and 1.1) and Heavy-REE at or below Chondrite values, showing refractory compositions relative to most Western Tethyan mantle rocks (e.g., Müntener et al. 2010, Kaczmarek and Müntener 2010). Serpentinites show Ce, U, and Pb enrichment as well as varying Ba, Rb, and Eu abundances, consistent with seawater serpentinisation of the host peridotite (e.g.,Seitz and Hart 1973, Burgath et al. 1997) (see electronic supplementary material ESM1, Fig. 1).

Basalts are homogenous and show flat REE with a slight Light-REE fractionation (La_N_/Sm_N_ = 0.51–0.73 and La_N_/Yb_N_ = 0.56–0.95), no Eu anomaly (Eu* = 0.94–1.04) (Fig. 5). The major and trace element composition indicates that these are typical N-MORB basalts of the Western Tethys (Vannucci et al. 1993; Kaczmarek et al. 2008; Montanini et al. 2008).

Mg-gabbros show a large variation in Cr (2200–200 ppm) and Ni (1600–200 ppm) (Fig. 4). Eu anomalies range from flat to positive (Eu* = 0.97–2.6), and incompatible elements (e.g., Na_2_O, P_2_O_5_, TiO_2,_ Light-REE) are lower than in Fe–Ti gabbros (Fig. 4, 5). The decrease in Mg# is correlated with a decrease in compatible elements (Ni, Cr, and Al_2_O_3_). Fe–Ti gabbros have low compatible elements (Ni < 160 ppm; Cr < 140 ppm), whereas P_2_O_5_ reaches 1.44 wt% (Fig. 4 d), which is consistent with the highest Y and REE concentrations of any sample, suggesting accumulation of apatite. Trace element patterns are enriched and Eu anomalies range from positive to negative (Eu* = 0.82–1.68) (Fig. 5).

### Mineral chemistry

The complete data set can be found in the electronic supplementary material (ESM2, Tables 1, 2, 3, 4).

**Table 3:** Radiogenic isotopic composition of bulk rocks (wr), clinopyroxene (cpx), and plagioclase (plg) of Civrari and Lanzo gabbros and MORB

Sample	Location	Sample type	Rb (ppm)	Sr (ppm)	^87^Sr/^86^Sr	± 1σ	Sm (ppm)	Nd (ppm)	^143^Nd/^144^Nd	± 1σ	^147^Sm/^144^Nd	^143^Nd/^144^Nd_160Ma_	eNd_160Ma_
MG-C1	Civrari	plg	0.02	178.31	0.702540	4	0.06	0.25	0.513106	21	0.155	0.512944	9.99
MG-C1	Civrari	cpx	0.00	7.07	0.702771	8	0.82	1.37	0.513292	2	0.359	0.512916	9.45
FG-EL1	Civrari	wr	1.09	134.10	0.702875	2	15.13	48.33	0.513117	1	0.189	0.512920	9.52
Pr2	Civrari	wr	10.72	170.77	0.703765	2	2.73	8.07	0.513132	1	0.203	0.512919	9.50
MG-C4	Civrari	wr	1.06	315.38	0.704308	2	0.83	2.21	0.513148	2	0.227	0.512911	9.34
FG-J4	Civrari	wr	1.15	21.46	0.703302	4	2.16	3.76	0.513161	3	0.345	0.512800	7.18
MG-J4	Civrari	wr	0.63	13.83	0.704784	11	2.88	7.34	0.513140	1	0.236	0.512893	9.00
MG-C1	Civrari	wr	0.99	100.70	0.702566	2	0.29	0.61	0.513218	3	0.291	0.512914	9.40
MG-89g	Civrari	wr	15.27	155.38	0.704412	2	2.32	6.95	0.513121	2	0.201	0.512911	9.34
Rog	Civrari	wr	0.90	130.71	0.704075	3	3.27	9.51	0.513127	1	0.207	0.512910	9.33
Mu5^a^	Lanzo	cpx	0.04	14.51	0.702646	5	1.18	2.49	0.513196	3	0.284	0.512898	9.09
Mu5^a^	Lanzo	plg	0.09	326.87	0.702658	6	0.08	0.36	0.512965	9	0.131	0.512828	7.72
Mu6^a^	Lanzo	cpx	b.d.	16.7	0.702494	28	0.93	1.89	0.513211	3	0.296	0.512901	9.16
Mu6	Lanzo	cpx	b.d.	16.2	0.702659	11	0.96	1.96	0.513220	2	0.296	0.512911	9.34
Mu6	Lanzo	cpx	b.d.	16.5	0.702709	6	0.94	1.88	0.513219	2	0.301	0.512903	9.19
Mu7^a^	Lanzo	cpx	0.02	17.2	0.702481	8	2.49	5.34	0.513205	2	0.280	0.512912	9.36
Mu7^a^	Lanzo	plg	0.07	415.7	0.702475	3	0.15	0.86	0.513022	20	0.102	0.512915	9.42

Sample	Location	Sample type	^206^Pb/^204^Pb	± 1σ	^207^Pb/^204^Pb	± 1σ	^208^Pb/^204^Pb	± 1σ	Pb ppm	U ppm	Th ppm
MG-C1	Civrari	plg	–		–		–		–	–	–
MG-C1	Civrari	cpx	–		–		–		–	–	–
FG-EL1	Civrari	wr	18.0792	17	15.4961	15	37.7177	35	0.610	0.117	0.122
Pr2	Civrari	wr	18.3647	14	15.5114	12	37.6774	30	0.570	0.110	0.097
MG-C4	Civrari	wr	18.4387	25	15.6055	22	38.2265	55	0.607	0.076	0.033
FG-J4	Civrari	wr	18.2367	40	15.5615	34	38.0665	83	0.514	0.028	0.027
MG-J4	Civrari	wr	18.4980	34	15.6699	31	38.5999	82	0.781	0.023	0.055
MG-C1	Civrari	wr	18.1414	64	15.5600	56	37.9529	137	0.209	0.007	0.008
MG-89g	Civrari	wr	18.2688	28	15.6138	25	38.2142	62	0.920	0.053	0.063
Rog	Civrari	wr	18.1230	14	15.5588	16	37.9366	55	1.001	0.067	0.115
Mu5^a^	Lanzo	cpx	–		–		–		–	–	–
Mu5^a^	Lanzo	plg	–		–		–		–	–	–
Mu6^a^	Lanzo	cpx	–		–		–		–	–	–
Mu6	Lanzo	cpx	–		–		–		–	–	–
Mu6	Lanzo	cpx	–		–		–		–	–	–
Mu7^a^	Lanzo	cpx	–		–		–		–	–	–
Mu7^a^	Lanzo	plg	–		–		–		–	–	–

^a^Leached samples

**Table 4:** Geothermometry of Civrari residual peridotites

	T_BKN_	T_WE–S_	T_W–W_	T_ca Opx_	T_V_	T_Sc_	T_Co_	T_REE_
*T* (°C)	1120	1120	1130	1240	1240	1320	1310	1380
1*σ*	75	35	63	35	18	27	35	47

T_Ca_ and T_BKN_ are the Ca-in-orthopyroxene thermometer and the two pyroxene thermometer of Brey and Köhler (1990), respectively; T_WE–S_ is the clinopyroxene–orthopyroxene thermometer of Witt-Eickschen and Seck (1991); T_W–W_ is the Webb and Wood (1986) thermometer based on the Cr/Al relationship of spinel and pyroxenes; T_V_, T_Sc_, and T_Co_ are pyroxene trace element thermometers from Seitz et al. (1999); and T_REE_ is the pyroxene REE thermometer from Liang et al. (2013)

### Ultramafic rocks

#### Olivine

Olivine is homogeneous within each sample. Both reactive and refractory olivine have an Mg# between 89.5 and 90.1. TiO_2_ abundances increase slightly from refractory olivine (0.002 wt%) to reactive olivine (0.003–0.005 wt%) as do heavy REE (e.g., Yb < 0.021 ppm to 0.021–0.037 ppm, respectively).

#### Spinel

**Fig. 6:**
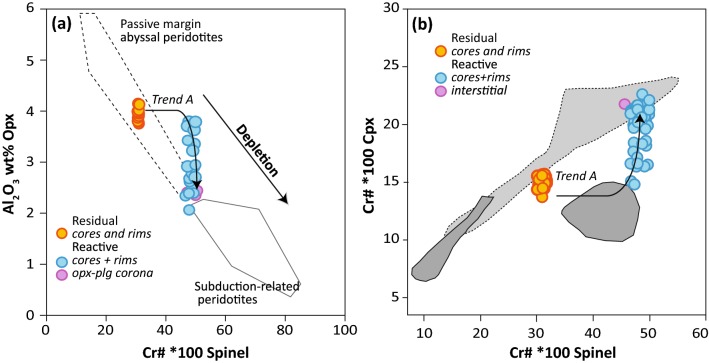
**a** Cr# in spinel vs Al_2_O_3_ wt% in orthopyroxene modified from Parkinson et al. (2003) with compositional fields from Bonatti and Michael (1989), showing the compositional evolution of spinel and orthopyroxene during partial melting. **b** Cr# spinel vs Cr# clinopyroxene. Dark grey area: spinel and plagioclase peridotites from the Eastern Central Alps (Müntener et al. 2010). Light grey area, dotted rim: spinel-peridotites from the Lena through (Hellebrand and Snow 2003). Trend A: compositional evolution of orthopyroxene, clinopyroxene and spinel during melt percolation

Cr-spinel is found as mm-size brown homogeneous anhedral grains or as small interstitial symplectites (Fig. 2b). Cr# [Cr/(Cr + Al + Fe^3+^) × 100] and TiO_2_ abundances increase from 30.2 to 52.5 and 0.06 to 0.31 wt% from the residual to the reactive peridotite, respectively, correlated with a concomitant decrease in Al_2_O_3_ in pyroxene (Fig. 6a, b). Civrari spinel have Cr# and Mg# that span the range of residual abyssal peridotites (Warren 2016) and TiO_2_ abundances (< 0.3 wt%) are consistent with low TiO_2_ of residual abyssal peridotites (Dick and Bullen 1984).

#### Clinopyroxene

Clinopyroxene is found as euhedral to anhedral grains. Orthopyroxene exsolution lamellae ≤ 5 micrometers along cleavage planes thin out at the rims. Interstitial clinopyroxene is intergrown with spinel, forming symplectitic textures. Textural observations indicate that clinopyroxene grain size varies more strongly in the sample with reaction textures, leading to smaller, rounder grains with rims of orthopyroxene–plagioclase intergrowths (Fig. 2a, b).

Refractory clinopyroxene shows some of the lowest Na_2_O (≤ 0.1 wt%) and TiO_2_ (≤ 0.13 wt%) contents ever measured in Western Tethys ophiolites and abyssal peridotites (e.g., McCarthy and Müntener 2015). Al_2_O_3_ abundances range from 4.4 to 5.2 wt% and Cr_2_O_3_ contents from 1.15 wt% to 1.4 wt%, whilst Mg# varies between 90 and 91.2, similar to depleted abyssal clinopyroxene compositions (Seyler et al. 2004, 2007, Warren and Shimizu 2010). Reactive clinopyroxene have homogenous Mg# (89.8–91.6) but are enriched in Na_2_O (0.25–0.40 wt%) compared to the residual peridotite. Reactive clinopyroxene core compositions overlap with residual clinopyroxene but show decreasing Al_2_O_3_ (5.3–3.0 wt%) and increasing TiO_2_ (0.08–0.2 wt%) and Cr# towards the rims (Fig. 6).

**Fig. 7:**
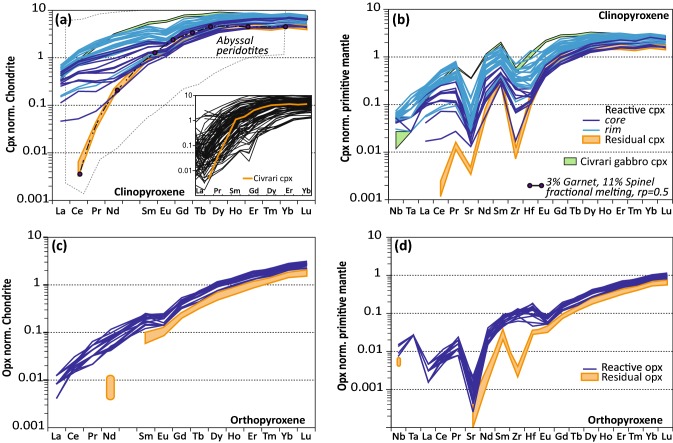
REE pattern (normalized to chondrite) and spider diagram (primitive-mantle normalized) of: **a**, **b** Civrari peridotite clinopyroxene; **c**, **d** Civrari peridotite orthopyroxene. Abyssal peridotites field from Warren, 2016. Primitive mantle and chondrite after McDonough and Sun (1995). Fractional melting modeling from McCarthy and Müntener (2015). *rp* residual porosity. In **a**, the inset corresponds to the comparison of depleted Civrari clinopyroxene (in orange) with the diversity of residual abyssal harzburgites compilation from Warren (2016)

**Fig. 8:**
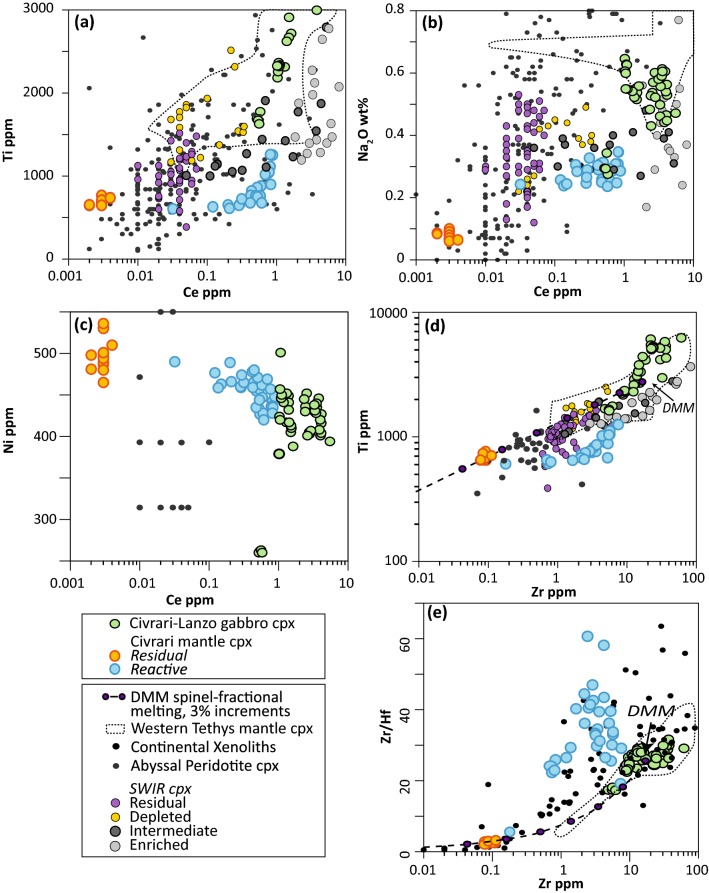
**a**–**c** Ce (ppm) enrichment in clinopyroxene during cryptic melt percolation vs Ti (ppm), Na_2_O (wt%) and Ni (ppm) in clinopyroxene. Note the rapid enrichment in Light-REE and Na prior to Ti enrichment and Ni depletion. **d** Zr (ppm) vs Ti (ppm) of mantle clinopyroxene during cryptic melt percolation; **e** Zr (ppm) vs Zr/Hf ratio of mantle clinopyroxene. Fractional melting modeling using starting modes and melting modes of Kinzler (1997), composition of DMM clinopyroxene from Workman and Hart (2005) and fractional melting equation from Johnson et al. (1990). Partition coefficients are from Suhr et al. (1998) and Hart and Dunn (1993), Kelemen et al. (1993) and Kelemen et al. (2003). Data for comparison: continental xenoliths are from Weyer et al. (2003), and Downes et al. (2003, 2015). Southwest Indian Ridge (SWIR) clinopyroxene and classification (residual, depleted, intermediate, and enriched) from Warren and Shimizu (2010). Field of Western Tethys clinopyroxene (spinel and plagioclase peridotites) from Müntener et al. (2004), Tribuzio et al. (2004), Rampone et al. (2008) and Müntener et al. (2010). Small grey circles correspond to the compilation of abyssal peridotites from Warren (2016) and Mid-Atlantic Ridge peridotites from Brunelli et al. (2006)

Trace element compositions of clinopyroxene range from homogeneous and strongly depleted (Ce_N_/Yb_N_ = ~ 0.001), consistent with depleted clinopyroxene from abyssal peridotites, to light-REE enriched with core-rim zonations (Ce_N_/Yb_N_ = 0.037–0.11) (Fig. 7a, b). Reacted clinopyroxene surrounded with orthopyroxene + plagioclase intergrowth display the highest Light-REE. Some reacted clinopyroxene cores show similar trace element patterns (e.g., Zr/Hf and Middle-REE) and major elements compositions as non-reacted refractory clinopyroxene (Fig. 7a, b). Abundances in Ce, Ti, Na_2_O, and Zr in clinopyroxene increase from residual to reactive peridotites (Fig. 8). In contrast, compatible elements such as NiO decrease in the reactive peridotite (Fig. 8). Zr/Hf ratios are low and near-homogenous for residual peridotites (2–3.2) but significantly higher and more heterogeneous for reacted clinopyroxene (5.6-60.7).

#### Orthopyroxene

Orthopyroxene is euhedral to subhedral. A second generation of orthopyroxene co-crystallizes with plagioclase and surrounds clinopyroxene (Fig. 2b). Orthopyroxene (Mg# 90.5–90.8) show high CaO (1.6–2.1 wt%) and low Al_2_O_3_ (3.7–4.2 wt%) and TiO_2_ contents (< 0.06 wt%), consistent with orthopyroxene from depleted abyssal peridotites (Seyler et al. 2004, 2007; Warren 2016) (Fig. 6a). The rims of reactive orthopyroxene show a decrease in Al_2_O_3_, reaching 2.0 wt% whilst preserving cores similar to refractory orthopyroxene (Fig. 6a). Orthopyroxene show strongly fractionated REE patterns, with typical Light-REE depleted patterns (Fig. 7 c,d) with reactive orthopyroxene and orthopyroxene–plagioclase coronas showing an overall increase in trace element abundances, a more pronounced negative Eu anomaly (Eu* = 0.49–0.70) and enrichment in Zr and Hf. Ce_N_/Yb_N_ for reactive orthopyroxene remains between 0.007 and 0.012 (Fig. 7 c, d).

### Gabbros

#### Clinopyroxene

**Fig. 9:**
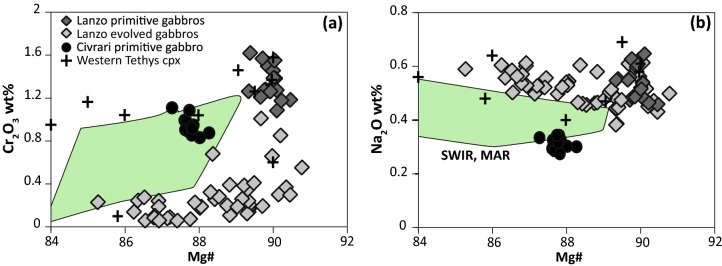
Composition of clinopyroxene from Civrari and Lanzo gabbros: **a** Mg# vs Cr_2_O_3_ (wt%) and **b** Mg# vs Na_2_O (wt%). Western Tethys clinopyroxene are from gabbros and troctolites from Rampone et al. (1998), Desmurs et al. (2002) and Montanini et al. (2008). Gabbro clinopyroxene are from the Southwest Indian Ridge (SWIR) and the Mid-Atlantic Ridge (MAR) (Coogan et al. 2001; Niu et al. 2002; Ross and Elthon 1997)

Subhedral clinopyroxene in the Monte Civrari gabbro are homogenous, and have elevated Cr_2_O_3_ (~ 0.95 wt%) and low Al_2_O_3_ (~ 3.5 wt%), TiO_2_ (~ 0.3 wt%) and Na_2_O (~ 0.3 wt%) with Mg# ranging from 86.9 to 87.5 (Fig. 9a, b). Primitive Lanzo gabbros have Cr-rich (1.4 wt%) subhedral green clinopyroxene, with an Mg# between 89.3 and 90.7. Al_2_O_3_ ranges between 3.35 and 5.52 wt% and both TiO_2_ (0.33–0.57 wt%) and Na_2_O (0.42–0.66 wt%) are slightly higher than for the Civrari gabbro (Fig. 9). More evolved Lanzo gabbros display euhedral Cr-poor, brown-reddish clinopyroxene with more evolved and heterogeneous compositions. Mg# varies between 90.5 and 84.8, whilst Cr, though low in cores (< 0.1 wt%) reaches up to 1 wt% in contact with the host peridotite. TiO_2_ shows normal zoning, from ~ 0.5 wt% in the cores to ~ 1.2 wt% at the rims, whereas Na_2_O is constant, between 0.4 and 0.6 wt% (Fig. 9). For further descriptions of zoning patterns and microprobe mapping of clinopyroxene see electronic supplementary material ESM1.

**Fig. 10:**
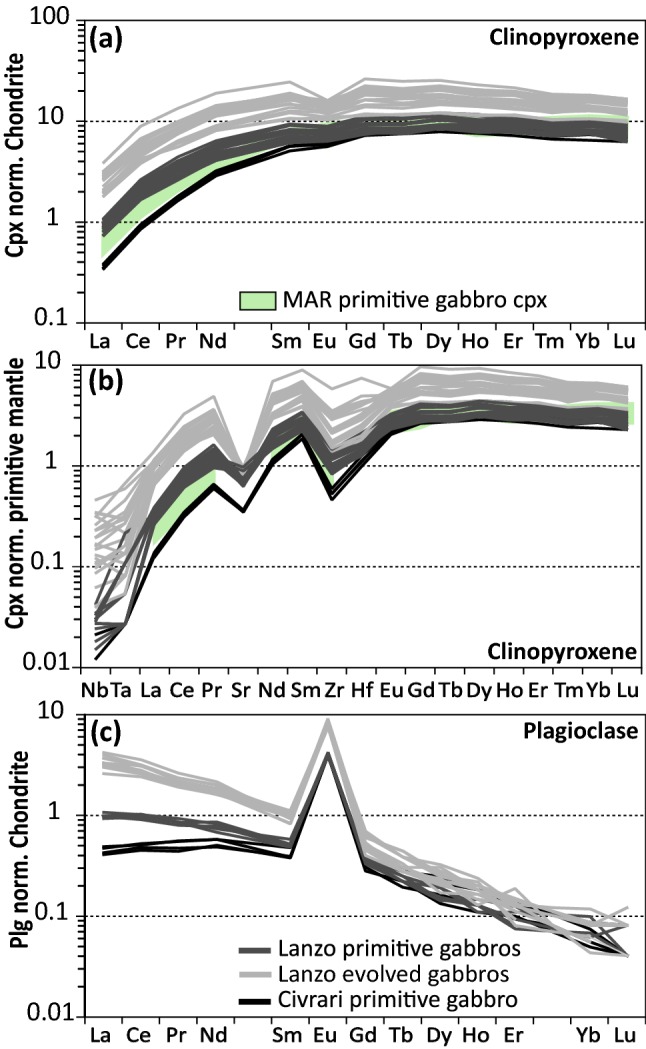
Trace element abundances of clinopyroxene and plagioclase from Civrari and Lanzo gabbros. **a** Clinopyroxene REE normalized to chondrite; **b** Clinopyroxene trace elements normalized to PM; **c** Plagioclase REE normalized to chondrite. Chondrite and PM from McDonough and Sun (1995). Mid-Atlantic Ridge (MAR) gabbro clinopyroxene from Coogan et al. (2001)

Trace element patterns of the Civrari gabbro clinopyroxene are more Light-REE depleted (La_N_/Sm_N_ = 0.078 and La_N_/Yb_N_ = 0.061) than primitive Lanzo gabbro clinopyroxene (La_N_/Sm_N_ = 0.122 and La_N_/Yb_N_ = 0.103). More evolved gabbros show an increase in REE and increasing negative Eu anomaly in clinopyroxene (Fig. 10a, b). Interstitial clinopyroxene in evolved Lanzo gabbros is enriched in REE, incompatible trace elements, and a pronounced depletion in Eu, consistent with crystallization of trapped melt fractions in equilibrium with plagioclase.

#### Plagioclase

Euhedral plagioclase from the Civrari gabbro ranges from An75 to An69, while the Lanzo gabbros have more evolved plagioclase, ranging from An64 to An49. Civrari plagioclase displays flat Light-REE pattern (La_N_/Sm_N_ between 1 and 1.2) and Lanzo gabbros show Light-REE enriched patterns, with La_N_/Sm_N_ between 1.7–2.0 and 3.1–4.5 for primitive and evolved gabbros, respectively (Fig. 10c).

#### Olivine

Olivine is anhedral and mostly altered. Civrari gabbro olivine is homogenous and have a Mg# between 86.2 and 86.7 and NiO concentration averaging 0.22 wt%.

### Radiogenic isotopes

**Fig. 11:**
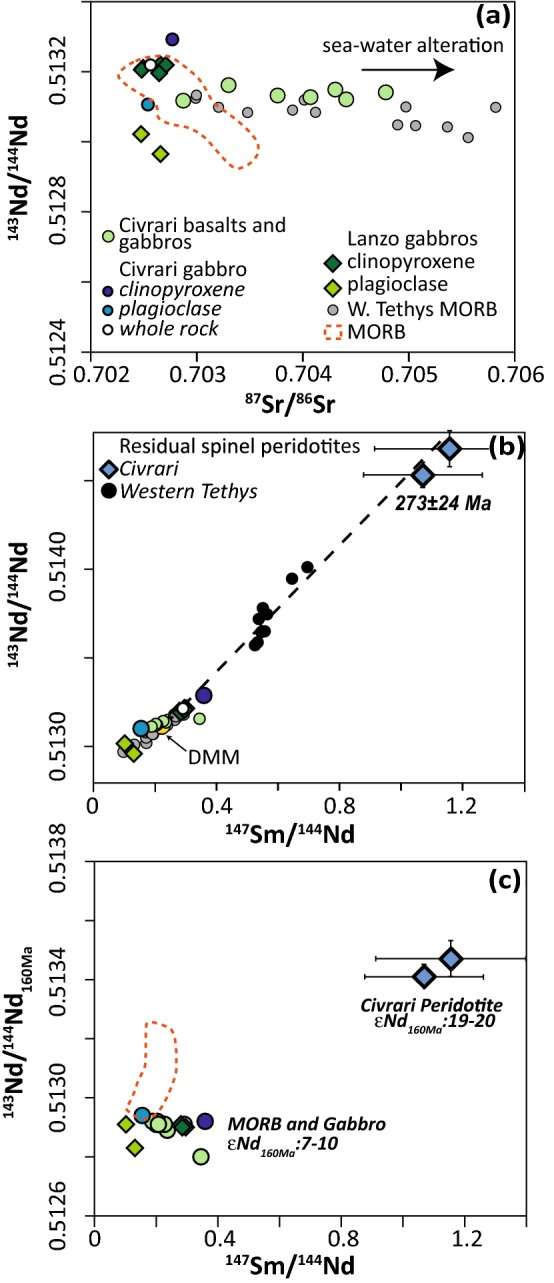
**a** Present-day ^87^Sr/^86^Sr vs ^143^Nd/^144^Nd of Civrari mafic rocks, clinopyroxene, and plagioclase of Civrari and Lanzo gabbros.; **b** ^147^Sm/^144^Nd vs present-day ^143^Nd/^144^Nd of Lanzo and Civrari gabbros. Black circles represent residual Alpine spinel-peridotites from the Western Tethys (data from Tribuzio et al. 2004; Rampone et al. 1996; Müntener et al. 2004). **c** ^147^Sm/^144^Nd vs age-corrected (160 Ma) ^143^Nd/^144^Nd of Lanzo and Civrari gabbros compared to the Civrari residual peridotites (data from McCarthy and Müntener 2015). The 160 Ma age corresponds to the age of MORB magmatism during opening of the Western Tethys (Piemont-Liguria ocean) (e.g., Tribuzio et al. 2016; Kaczmarek et al. 2008). Tethyan MORB includes gabbros and basalts from the Internal Liguride (Rampone et al. 1998), Platta (Schaltegger et al. 2002), External Liguride (Tribuzio et al. 2004) and Montgenèvre Ophiolite (Costa and Caby 2001). MORB basalts are from White and Hofmann (1982) and Ito et al. (1987)

Measured whole-rock ^143^Nd/^144^Nd of gabbros vary between 9 and 10 εNd_160Ma_, similar to Western Tethyan Jurassic gabbros and MORB (Rampone et al. 1996, 1998; Schaltegger et al. 2002; Tribuzio et al. 2004) (Fig. 11). One gabbro sample (FG-J4) dominated by clinopyroxene + Fe–Ti oxides, displays slightly lower εNd (εNd_160Ma_ =+ 7.18). ^87^Sr/^86^Sr preserves less radiogenic MORB-like magmatic values in less altered gabbros and mineral separates whereas seawater alteration has affected the ^87^Sr/^86^Sr of most bulk rocks leading to a sharp increase in ^87^Sr/^86^Sr at constant ^143^Nd/^144^Nd (McCulloch et al. 1981) (Fig. 11). Pb isotope ratios are similarly affected by seawater alteration but less altered bulk samples show Pb-isotopic signatures similar to MORB (Table 3 and electronic supplementary appendix ESM1, Fig. 3). Age-corrected εNd_160Ma_ (7–10) of Civrari basalts and gabbros are not in isotopic equilibrium with spatially associated Civrari peridotites (εNd_160Ma_ of 19–20) (McCarthy and Müntener 2015) (Fig. 11).

## Discussion

### Residual nature of Civrari peridotites and ancient melting events

Low bulk Al_2_O_3_ and TiO_2_ abundances highlight the large-scale homogeneity and residual nature of the Civrari ultramafic massif (Fig. 3). This contrasts sharply with the compositional heterogeneity of Western Tethys peridotites from the European Alps and Apennines and more closely resembles the compositional spectrum of residual abyssal peridotites. The depleted bulk composition of Civrari serpentinites and peridotites (Figs. 3, 5a, b) is the result of fractional melting. The Cr# and TiO_2_ abundances of spinel and low Na_2_O contents and strongly fractionated REE trends in clinopyroxene (Figs. 7, 8) are consistent with a residual signature after ca. 3% melting in the garnet stability field and ca. 10–11% melting in the spinel stability field (McCarthy and Müntener 2015). Local microtextures of interstitial clinopyroxene-spinel ± olivine symplectites indicating low-volume melt retention in the stability field of spinel.

Whether or not the Civrari peridotites represent residues of near-fractional melting related to the spatially associated basalts and gabbros remains controversial. Several residual spinel-peridotites from the Western Tethyan mantle are not in isotopic equilibrium with spatially associated lavas (e.g., Rampone et al. 1998; Müntener et al. 2004), as also illustrated by Civrari peridotites and associated magmatism (Fig. 11b, c). One hypothesis is that the Tethyan upper mantle is isotopically heterogeneous, which leads to a geochemically heterogeneous oceanic lithosphere (e.g., Cipriani et al. 2004; Sanfilippo et al. 2019). This hypothesis is supported by mantle heterogeneity along modern mid-ocean ridge systems (e.g., Stracke et al. 2011; Warren et al. 2009). An alternative hypothesis suggests that mantle heterogeneity is inherited from the subcontinental lithosphere (McCarthy and Müntener 2015; Rampone et al. 1998; Müntener et al. 2004). Indeed, parts of the western Tethyan ophiolites are derived from former ocean–continent transition zones (e.g., Müntener et al. 2010; Manatschal & Müntener 2009). Thus, rift-related extension leads to the exhumation of subcontinental mantle with inherited ancient melting and metasomatic imprints, locally erased by refertilisation during incipient opening of the Western Tethys (e.g., Picazo et al. 2016). This alternative interpretation is also supported by the presence of cold (850–1050 °C) spinel-harzburgites affected by high-degree partial melting (14–25%) along the magma-poor Newfoundland ocean-continent transition zone (Müntener and Manatschal 2006). Depleted spinel-peridotites, found throughout the Alpine-Apennine orogen show a coherent Nd-isotopic “errorchron” at 273 ± 24 Ma (McCarthy and Müntener 2015) (Fig. 11b). This isotopic trend over a large geographical region is surprising but consistent with widespread Permian-age mafic magmatism throughout Western Europe (e.g., Pin 1986; Schaltegger and Brack 2007). This might be a coincidence, but supports the alternative interpretation that isotopic disequilibrium in western Tethys ophiolites is related to inherited, ancient melting events.

Despite evidence for melt metasomatic processes in the subcontinental mantle worldwide (Wiechert et al. 1997; Witt-Eickschen and Kramm 1998; Downes 2001; Ionov et al. 2002), the homogeneous composition of Civrari peridotites and serpentinites indicates that km-scale subcontinental mantle domains may remain unaffected by metasomatic processes for over 100 Ma. Depleted characteristics of peridotites in (ultra)slow-spreading systems therefore cannot be used to discount a subcontinental mantle origin (e.g., Lassiter et al. 2014).

### Rapid cooling of high-temperature Civrari peridotites

Equilibrium temperatures for the refractory spinel peridotite at 1.5 GPa are reported in Table 4. Geothermometers based on major elements have lower calculated temperatures (1120–1240 °C) than thermometers calculated using trace elements (1240–1380 °C) (Table 4). The T_REE_ of Liang et al. (2013), based on slow diffusion elements, gives the highest calculated *T*°, reaching 1380 ± 47 °C, which is within the range of the dry peridotite solidus of ca. 1300–1350 °C at 1.5–2 GPa (Hirschmann 2000). These elevated temperatures are consistent with elevated CaO in orthopyroxene (~ 2 wt%), favouring the interpretation that Civrari peridotites represent a rare case of near-fractional melting (Fig. 7a). These elevated temperatures are unlike those estimated for other Western Tethys mantle peridotites, which range from 850 to 1100 °C (Rampone et al. 1996; Rampone et al. 2005; Müntener et al. 2010).

The comparison of T_BKN_ and T_REE_ indicates fast cooling rates similar to abyssal peridotites and significantly faster than cooling rates recorded by subcontinental mantle peridotites along the Western Tethys (Dygert and Liang 2015). The Civrari peridotites thus represent either rapid cooling of an abyssal peridotite at an active spreading ridge, or, alternatively, implies rapid cooling of subcontinental lithosphere. As discussed previously, we interpret the isotopic decoupling between western Tethyan mantle and mafic rocks as caused by ancient (273 ± 24 Ma) near-fractional melting unrelated to Jurassic MOR-type magmatism (Fig. 11b, c). The Civrari peridotites therefore record high-temperatures and rapid cooling as a result of rapid exhumation from a deeper, warmer section of the subcontinental mantle (e.g., Picazo et al. 2016).

### Low-pressure MORB crystallization in exhumed subcontinental mantle

The most enriched mantle clinopyroxene evolve towards the compositional field of clinopyroxene from gabbroic dykes (Figs. 7, 8). This is illustrated by the enrichment of Ce, Ti, Zr and Na_2_O as well as decreasing Ni of reactive mantle clinopyroxene towards the composition of gabbroic clinopyroxene (Fig. 8). We argue that the melt percolating through and reacting with the Civrari peridotite was of similar composition as the spatially associated gabbros. Pb isotopic composition (see electronic supplementary appendix ESM1, Fig. 3), unradiogenic ^87^Sr/^86^Sr (< 0.703), and radiogenic Nd (εNd = 7–10) of unaltered whole-rocks and mineral separates indicate MORB-like isotopic compositions (Fig. 11). This is also consistent with the bulk composition of Civrari basalts, which are similar to mid-ocean ridge basalts (Fig. 4). Clinopyroxene compositions from gabbros and reactive clinopyroxene resemble clinopyroxene from mid-ocean ridge gabbros (e.g., Coogan et al. 2001) (Figs. 9, 10a, b). Thus, isotopic compositions, mineral chemistry and bulk rock chemistry indicate that ascent of MORB-melts leads to the emplacement of gabbroic dykes and localized melt percolation during cooling of the peridotites.

The compositional variation of Civrari gabbros mirrors the range found in other Tethyan ophiolites (Desmurs et al. 2002; Kaczmarek et al. 2008; Montanini et al. 2008) (Fig. 4). Most Mg-gabbros have positive Eu, Sr anomalies and high Al_2_O_3_ contents suggesting crystal fractionation dominated by plagioclase whereas gabbros with lower Al_2_O_3_ and increasing Cr and Ni contents (Fig. 4) suggest accumulation of clinopyroxene (± spinel) and olivine, respectively. Bulk rock gabbro compositions show compositional trends similar to fractionating MORBs (Figs. 4, 5b), with increasing Zr, P_2_O_5_, and TiO_2_ with decreasing Mg#. Textures from the Civrari gabbro dyke indicate a crystallization sequence of olivine > plagioclase > clinopyroxene, consistent with low-pressure evolution of MORB melts (< 5kbar) (Grove et al. 1993). Olivine, clinopyroxene and plagioclase compositions are comparable to primitive gabbros crystallized at 3-5 kbar in the Mid-Cayman Rise (Elthon et al. 1992; Ross and Elthon 1997) (Fig. 9). Jurassic gabbros and basalts from the Western Tethys plot along a low-pressure fractionation trend favouring enrichment of TiO_2_ and FeO in MORB (e.g., Villiger et al. 2007), leading to late-stage accumulation of ilmenite, titanomagnetite, apatite, and the formation of evolved cumulates with high TiO_2_ and P_2_O_5_ (Fig. 4).

Euhedral clinopyroxene from Lanzo gabbros has higher Mg# and Al_2_O_3_, suggesting slightly higher pressure of crystallization (ca. 5kbar) (Grove et al. 1993; Langmuir et al. 1992; Feig et al. 2006) (Fig. 9b). Increasing pressure will lead to a suppression of plagioclase and olivine primary phase fields and an expansion of the clinopyroxene field (Presnall et al. 1978; Sen and Presnall 1984), leading to clinopyroxene with higher Al_2_O_3_ and Cr_2_O_3_ and less-pronounced negative Eu anomalies as well as higher NiO. Primitive Lanzo clinopyroxene shows less fractionated Light-REE and higher Na_2_O abundances as well as higher La/Sm ratios in plagioclase compared to Civrari gabbro clinopyroxene and plagioclase (Figs. 9, 10). Similar isotopic compositions (Fig. 11) indicate that the compositional variability of Civrari and Lanzo gabbros results primarily from slight differences in the degree of partial melting of a depleted mantle source.

### Cryptic grain-scale infiltration of MORB-melts

The reactive harzburgite shows distinct microtextures indicative of melt–rock interaction coupled with high MgO and low SiO_2_ implying high olivine abundances. Primary MORB-type melts originating from partial melting of peridotite at 10–30 kb will be saturated in olivine + Al-phase (Kelemen 1990) leading to pyroxene dissolution and olivine crystallization, forming reactive high-MgO mantle rocks. Si-saturation of ascending melt will then lead to the dissolution of clinopyroxene and crystallization of orthopyroxene + plagioclase in the plagioclase stability field, consistent with the presence of orthopyroxene + plagioclase intergrowth around rounded, anhedral mantle clinopyroxene and spinel grains (Fig. 2b). TiO_2_ in spinel is correlated with increasing Cr#, consistent with reaction of a MORB-melt and crystallization of plagioclase (Dick 1989). However, even though reacted clinopyroxene show higher Na_2_O concentrations and higher Cr# in spinel, consistent with equilibrium with plagioclase (e.g., Müntener et al. 2010), they retain a refractory composition compared to other clinopyroxene from Western Tethyan ultramafic rocks (Fig. 8). This indicates that melt percolation at a local-scale is not as pervasive as generally recorded in Western Tethys ophiolites (Piccardo et al. 2007; Müntener et al. 2010). On the scale of a thin sections (2.2 × 3.7 cm), reactive clinopyroxene varies from mm-size subhedral grains to rounded ~ 150 μm grains rimmed by orthopyroxene–plagioclase intergrowth ± apatite (Fig. 2d), indicating localized melt percolation and crystallization. The lack of equilibration of the peridotite with ascending melts is indicated by core-rim zonation in major and trace elements in mantle pyroxenes (Figs. 6, 7, 8). The presence of 3–5 μm apatite suggests trapped melt crystallization leading to apatite saturation due to increasing P_2_O_5_ in residual melt fractions. Fractional crystallization calculations at 4 kbar using Rhyolite-MELTS (Gualda et al. 2012) with a primitive basalt HK #19 (Hirose and Kushiro 1993) and 0.08 wt% P_2_O_5_ and 0.1 wt% H_2_O indicates that apatite saturation in MORB-type melts occurs just below 1100 °C, which provides an upper bound for ambient mantle temperature during melt entrapment.

Grain-scale melt percolation lead to well developped core-rim zonation patterns in clinopyroxene (Figs. 7, 8). The enrichment trend of reacted clinopyroxene overlaps the field of both residual and reactive clinopyroxene from abyssal peridotites (Warren and Shimizu 2010) (Fig. 8) and illustrates how sensitive Na_2_O and Light-REE are to local interactions with percolating melts. As can be seen in peridotites from the SWIR identified as residues after partial melting (Warren and Shimizu 2010) Na_2_O varies by a factor of five with no concomitant change in Ce (Fig. 8b). This indicates that these residual abyssal peridotites have been affected by localized, small-scale melt percolation or melt retention (e.g., Warren 2016).

Abyssal peridotites showing elevated Cr# in spinel, elevated Na_2_O abundance and enrichement in light-REE compared to Civrari residual peridotite cannot be readily explained by near-fractional melting. These chemical trends offer further support indicating that most residual abyssal peridotites show weak refertilisation and/or that most peridotites are not solely a result of near-fractional melting but retain significant amounts of melt upon decompression (Elthon 1992; Hellebrand et al. 2002; Brunelli et al. 2006; Warren and Shimizu 2010; Warren 2016). This demonstrates the importance of Na_2_O concentrations in clinopyroxene prior to determining their residual character, as clinopyroxene is prone to rapidly reequilibrate with diffuse, low-volume melts (e.g., Lundstrom 2000).

### Fractionating Zr–Hf ratios in mantle clinopyroxene: near-fractional melting and cryptic MORB-melt percolation

Civrari mantle clinopyroxene shows strongly variable Zr/Hf ratios (1–60) (Fig. 8e). These variations contrast with the proposal that Zr/Hf ratios are uniform and chondritic (Jochum et al. 1986). The Zr/Hf ratios of Civrari residual and refractory clinopyroxene overlap with the Zr/Hf ratios of subcontinental mantle xenoliths (Fig. 8e) (Lenoir et al. 2000; Downes et al. 2015). Civrari clinopyroxene show low Zr/Hf ranging between 2.1 and 3.2 and are the lowest ratios recorded in Western Tethys ophiolites (e.g., Müntener et al. 2010) and much lower than Chondrite (ca. 34.2, Jochum et al. 1986). These values are consistent with fractional melting modeling in the spinel stability field of an initial Depleted MORB Mantle (DMM) (Workman and Hart 2005) (Fig. 8e). Strongly subchondritic to superchondritic Zr/Hf (e.g., 0.5–82), high-depletion of Zr and Hf and elevated εHf at moderate εNd have been documented in xenoliths derived from the subcontinental mantle of Western Europe (Wittig et al. 2006; Downes et al. 2015). To the contrary of those samples that show enriched Light-REE implying mantle metasomatic processes, the Civrari residual peridotites were not affected by metasomatism. This implies that Zr/Hf fractionation results from near-fractional melting as a consequence of a slight difference in compatibility of Zr and Hf during mantle melting (e.g., David et al. 2000; Weyer et al. 2003; Stracke et al. 2011).

Cryptic percolation of MORB-melt in the reactive peridotite leads to significant variations in Zr/Hf ratios (20–60) at near-constant Zr at the grain scale (Fig. 8e). Zr/Hf ratios are significantly more variable, and Zr generally lower, than coeval clinopyroxene from intruding gabbros and from refertilised plagioclase peridotites (Müntener et al. 2010). As reacted clinopyroxene show enrichment trends evolving towards the composition of coeval MORB gabbro clinopyroxene (e.g., Figs. 7a, 8c) the variation in Zr/Hf in the case Civrari peridotites cannot be related to reaction of peridotites with exotic (e.g., carbonatite) melts, as proposed by Dupuy et al. (1992), Rudnick et al. (1993) and Downes et al. (2003) for intra-plate magmatism and metasomatised mantle xenoliths. The main distinction between refertilised plagioclase peridotites and reactive Civrari peridotites is related to the significant change in melt/rock ratio upon melt percolation. Müntener et al. (2010) calculated that up to 12% MORB-melt is retained during pervasive refertilization. This elevated melt/rock ratio is consistent with the Zr/Hf of these clinopyroxene overlapping the composition of gabbroic clinopyroxene (Fig. 8e). On the contrary, the textural and compositional variation of Civrari reactive clinopyroxene at the grain scale implies significantly lower melt/rock ratios. Thus, percolation of MORB-type melts at low melt/rock ratio fractionates Zr/Hf as a result of slight differences in compatibiliy of Zr and Hf (e.g., Navon and Stoler 1987; Bodinier et al. 1989). Cryptic percolation of MORB-melts upon rifting and mantle exhumation in ocean–continent transitions might lead to compositional variations in trace elements crucial for the long-term compositional evolution of the mantle lithosphere.

**Fig. 12:**
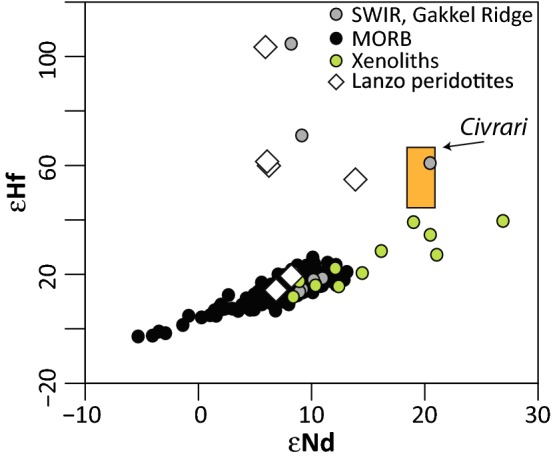
εNd vs εNf of MORB (Gale et al. 2013), Gakkel Ridge and Southwest Indian Ridge Peridotites (Stracke et al. 2011) and mantle xenoliths (Byerly and Lassiter 2014). Lanzo peridotites (western Tethys) are from Sanfilippo et al. (2019). Predicted field of εHf of Civrari is in orange; εNd values are from McCarthy and Müntener (2015). Note that these are present-day values, except for Civrari peridotites and Lanzo peridotites, which have been corrected to 160 Ma

Clinopyroxene with elevated radiogenic Hf signatures (εHf_160Ma_: 14–213) at MORB-like εNd (6–15) have been described in reactive harzburgites in western Tethys ophiolites (Southern Lanzo Massif, Sanfilippo et al. 2019). One possibility is that this isotopic signature is reflecting melting of “ultra-depleted” asthenospheric domains. However, western Tethys ophiolites have significant isotopic and petrological heterogeneities at small and large scale (e.g., Rampone and Hofmann 2012; Piccardo et al. 2007). Therefore, an alternative is that MORB-melts might have percolated into heterogeneous, subcontinental mantle with radiogenic Hf–Nd isotopic signatures inherited from previous melting and metasomatic events. MORB-melt percolation would equilibrate Nd isotopic ratios to MORB values more efficiently than Hf isotopic ratios (Stracke et al. 2011). The analysis of Hf-isotopic ratios of depleted western Tethys spinel-peridotites unaffected by subsequent melt percolation would allow to test these competing hypothesis. For example, assuming that the Civrari peridotite was in equilibrium with a DMM at 270 Ma upon partial melting (McCarthy and Müntener 2015), we predict, using the average and 2*σ* of Lu and Hf abundances of Civrari clinopyroxene and known DMM compositions (Workman and Hart 2005; Salters and Stracke 2004), that the measured ^176^Hf/^177^Hf should be within a 2*σ* range of εHf = 81–121 (average of εHf = 94–96). This implies a εHf_160Ma_ = 47–64 (average of 52–54), distinctly different than the εNd_160Ma_ of 19–20 (McCarthy and Müntener 2015). Such elevated εHf_160Ma_ abundances would be well within the range of εHf of mantle peridotites from the Gakkel Ridge, mantle xenoliths and reactive harzburgites from the western Tethys (Stracke et al. 2011; Byerly and Lassiter 2014; Sanfilippo et al. 2019; Downes et al. 2015) and significantly more radiogenic than MORB (Fig. 12).

## Conclusions

The Civrari peridotite shows similar petrological and geochemical characteristics as residual abyssal peridotites. Mantle clinopyroxene shows some of the most fractionated REE and lowest Na_2_O ever measured in abyssal peridotites and imply that these peridotites are the product of near-fractional melting starting in the garnet stability field. Melt percolation of MORB-melts similar in composition to coeval gabbroic intrusions lead to grain scale melt–rock interaction and induced the compositional zonation of reacted mantle clinopyroxene. We show that diffuse–melt percolation affects Na_2_O abundances of mantle clinopyroxene. The high *T*° (1200–1300 °C) recorded by these peridotites and the preservation of grain-scale compositional zonations are the consequence of rapid cooling and exhumation to the seafloor during Jurassic extension. The lack of isotopic equilibrium between mafic rocks and mantle peridotite suggests that partial melting is ancient and unrelated to MORB-type magmatism. Therefore, though Civrari peridotites are compositionally unlike other mantle rocks from the Western Tethys and show key characteristics of abyssal peridotites, such as km-scale homogeneity, a depleted nature, elevated recorded temperatures and fractional melting with limited melt percolation, they are the product of protracted magmatic processes occurring during Jurassic rifting superimposed over ancient (Permian) melting events.

## Electronic supplementary material

Below is the link to the electronic supplementary material.

Supplementary material 1 (PDF 2417 kb)

Supplementary material 2 (XLSX 154 kb)
