# Supplementary material for: Evidence for ancient fractional melting, cryptic refertilization and rapid exhumation of Tethyan mantle (Civrari Ophiolite, NW Italy)
Source: Contrib Mineral Petrol. 2019 Aug 1;174(8):69. doi: 10.1007/s00410-019-1603-5 (PMC6675762; doi:10.1007/s00410-019-1603-5)
Supplement: Supplementary file 1 — Supplementary material 1 (PDF 2417 kb) [file 410_2019_1603_MOESM1_ESM.pdf]

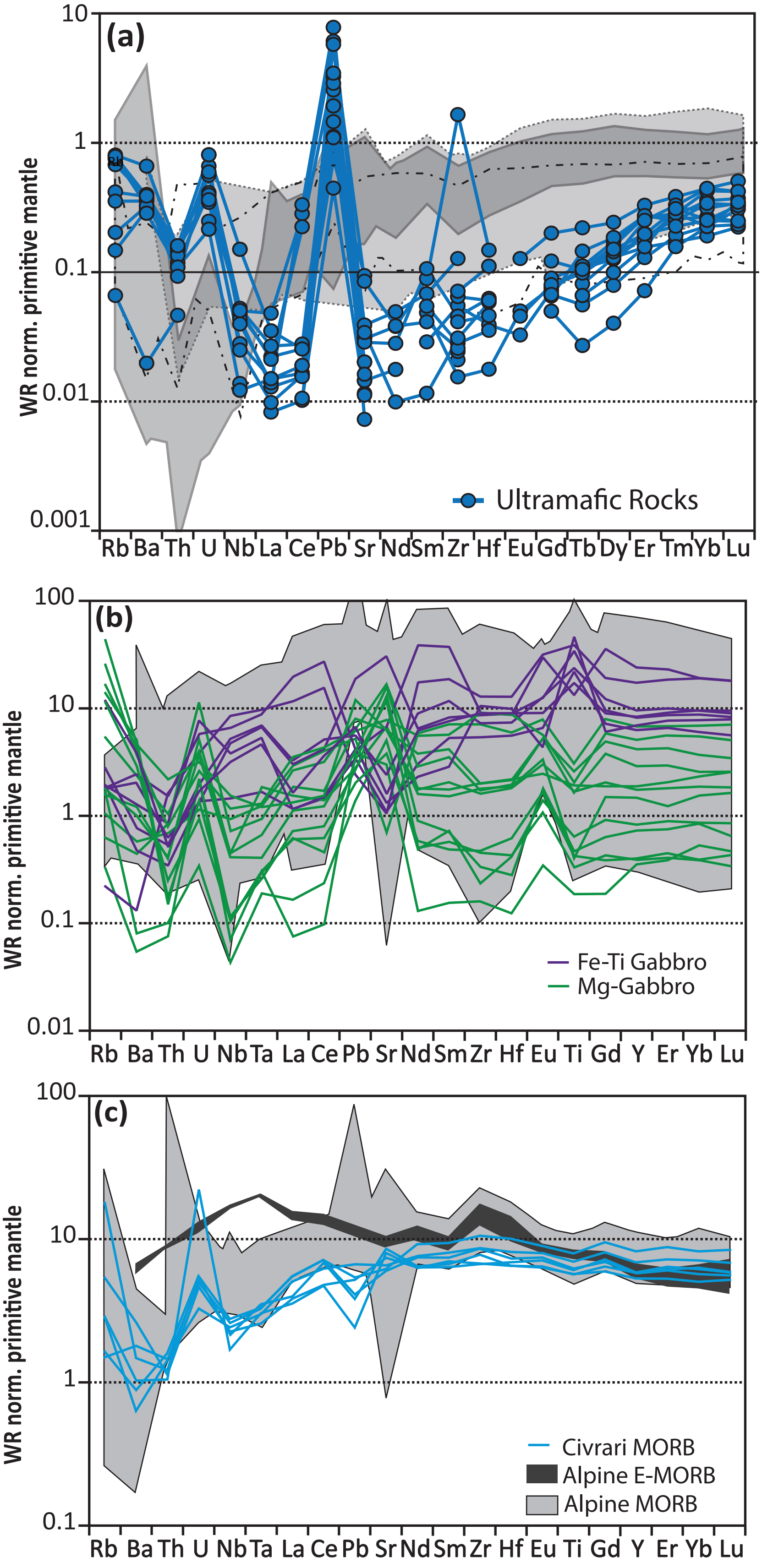

Figure 1: a) Spiderdiagram of whole-rock (WR) Civrari ultramafic rocks and compared to compositional fields of: Lanzo (continous black line), Malenco (large dotted line) and Platta (fine dotted line) rocks. Lanzo data from Kaczmarek & Müntener (2010), Malenco and Platta data from Müntener et al. (2010). b-c) Spiderdiagram of gabbros and basalts from Civrari; Data for comparison: E-MORB from Desmurs et al., (2002). Alpine N-MORB from Rampone et al., (1998), Desmurs et al., (2002), Montanini et al., (2008), Kaczmarek et al., (2008) and Chalot-Prat (2005). Chondrite and Primitive Mantle from McDonough & Sun (1995). (references can be found in the main text)

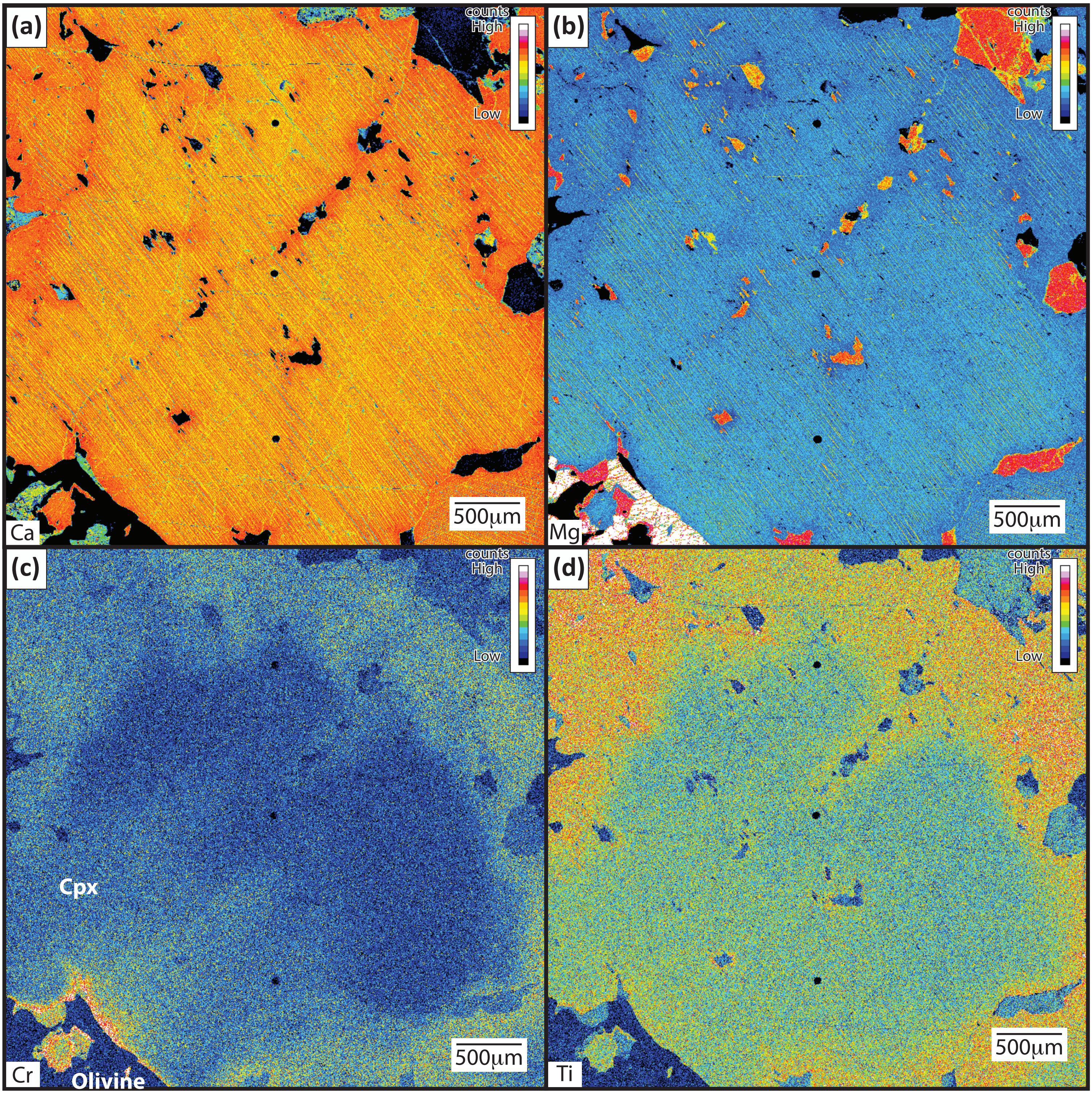

Figure 2: Mu8 microprobe map of clinopyroxene (cpx) phenocryst (Ca, Mg, Cr and Ti). Analytical conditions are 15kv, 20nA, 1micron beam size. This sample shows reverse zoning in Cr in clinopyroxene. Cr enrichement is coupled with increasing Ti, suggesting that the zonation is due to magmatic processes during crystallisation. On the other hand, very thin Cr-rich cpx rims showing no measurable enrichement in Ti (bottom left) are found nearest the host peridotite, suggesting subsolidus reequilibration after the crystallization of the gabbro dykelet. The slight chemical heterogeneity of these clinopyroxene is likely a reflection of in-situ crystallization within the host peridotite.

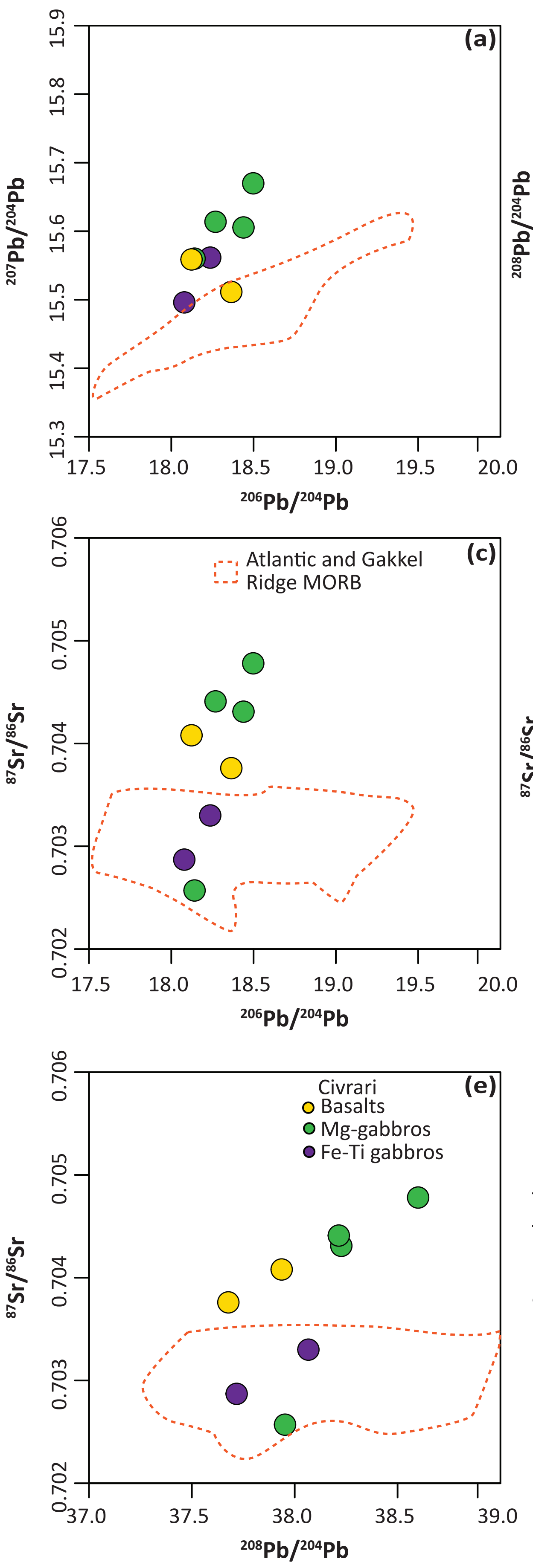

Figure 3: Pb-isotope variations in bulk rocks (gabbros and basalts) from the Civrari ophiolite. Field of Mid ocean ridge basalts (MORB) from the Mid-atlantic ridge and Gakkel ridge from Gale et al., (2013). Pb- and Sr isotope ratios are not age-corrected due to ocean alteration. Note that unaltered gabbros and Fe-Ti gabbros show Sr-Pb isotopic ratios overlapping with the field of MORB basalts.
